# RAS Inhibitor RMC‐7977 Blocks Vascular Overgrowth of *NRAS^Q61R^* Mutant Endothelial Cells

**DOI:** 10.1111/jcmm.71316

**Published:** 2026-08-10

**Authors:** Sara Alharbi, Svatava Merkle, Patricia Pastura, C. Griffin McDaniel, George S. Zaky, Andrew M. Waters, Timothy D. Le Cras

**Affiliations:** ^1^ Department of Cancer Biology University of Cincinnati College of Medicine Cincinnati Ohio USA; ^2^ Department of Pediatrics University of Cincinnati College of Medicine Cincinnati Ohio USA; ^3^ Division of Pulmonary Biology Cincinnati Children's Hospital Medical Center Cincinnati Ohio USA; ^4^ Division of Hematology Cincinnati Children's Hospital Medical Center Cincinnati Ohio USA; ^5^ Department of Surgery University of Cincinnati College of Medicine Cincinnati Ohio USA

**Keywords:** complex lymphatic malformation, HRAS, kaposiform lymphangiomatosis, KRAS, NRAS, vascular anomalies

## Abstract

RAS mutations occur in patients with several types of vascular anomalies, but effective treatments remain limited. To address this need, we evaluated the RAS (ON) multi‐selective inhibitor RMC‐7977 in human endothelial cells (ECs) expressing the NRAS^Q61R^ mutation found in kaposiform lymphangiomatosis (KLA). RMC‐7977 was evaluated using in vitro and in vivo models. Doxycycline‐inducible NRAS^WT^ and NRAS^Q61R^ human ECs were treated with RMC‐7977 (3.12–100 nM) or vehicle. We assessed signalling pathways, proliferation, migration, morphology, and angiopoietin‐2 (ANG‐2) production. NRAS^Q61R^ ECs in a 3D angiogenesis assay were also treated with RMC‐7977. For in vivo studies, NRAS^Q61R^ ECs were injected into flanks of nude mice on a doxycycline diet to generate xenografts. Mice received oral RMC‐7977 or vehicle, and xenografts were collected after 11 days. RMC‐7977 inhibited NRAS^Q61R^‐induced ERK phosphorylation and reduced proliferation, migration, spindle‐like morphology, and ANG‐2 production in a dose‐dependent manner. RMC‐7977 reduced vascular area in the angiogenesis assay. In vivo, RMC‐7977 reduced xenograft weight, vascular area, and p‐ERK staining. Overall, RMC‐7977 suppressed NRAS^Q61R^‐mediated signalling, aberrant EC behaviour, and ANG‐2 production in vitro and reduced vascular overgrowth in angiogenesis assays and mouse xenografts. Therefore, RMC‐7977 may be a promising therapeutic candidate for RAS‐driven vascular anomalies, including KLA.

**Abbreviations**
ANG‐2: angiopoietin‐2 protein
*ANGPT2*
: angiopoietin‐2 gene
AVMs: arteriovenous malformations
*CBL*
: casitas B lineage lymphoma gene
CCLA: central conducting lymphatic anomaly
CLAs: complex lymphatic anomalies
EBM2: endothelial cell growth medium‐2
ECs: endothelial cells
EPCs: endothelial progenitor cells
FBS: fetal bovine serum
GAP: GTPase‐activating proteins
GDP: guanosine diphosphate
GLA: generalized lymphatic anomaly
GSD: Gorham‐Stout disease
GTP: guanosine triphosphate
H: hours
HDLECs: human dermal lymphatic endothelial cells
*HRAS*
: Harvey rat sarcoma virus gene
HUVECs: human umbilical‐vein endothelial cells
Hydroxyurea: Hu
ISSVA: International society for the study of vascular anomalies
KHE: Kaposiform hemangioendothelioma
KLA: kaposiform lymphangiomatosis
*KRAS*
: Kristen rat sarcoma virus gene
LECs: lymphatic endothelial cells
MAPK: mitogen‐activated protein kinase
*NRAS*
: neuroblastoma rat sarcoma virus gene
*NRAS* ^Q61R^
: *NRAS p*.Q61R mutant gene
*NRAS* ^WT^
: *NRAS* Wild‐type gene
PI3K: phosphoinositide 3‐kinase
*RAF*
: rapidly accelerated fibrosarcoma gene
*RAS*
: rat sarcoma virus gene
VAs: vascular anomalies
VMs: vascular malformations

## 1 Introduction

Vascular anomalies (VAs) are broadly categorized into three major groups: vascular tumours, vascular malformations (VMs), and potentially unique vascular anomalies, exhibiting distinct clinical and pathological features, according to the 2025 updated classification from the International Society for the Study of Vascular Anomalies (ISSVA) [1]. VMs are a heterogeneous group of disorders characterized by malformed blood vessels, which may involve arteries, veins, lymphatic vessels, capillaries, or mixed vessel types [2]. VMs are further classified according to the developmental anomalies of specific vessel types and their haemodynamic properties, being divided into fast‐flow and slow‐flow types [1]. Genetic testing of patient biopsies, affected tissue, and circulating cell‐free DNA has revealed that many VMs arise from somatic gene mutations that hyperactivate components of the RAS/RAF/MEK or PI3K/AKT/mTOR signalling pathways [3, 4, 5, 6]. Despite growing insights into these genetic drivers, therapeutic options for patients with VMs remain limited, underscoring the need for improved targeted treatments.

The rat sarcoma virus (RAS) gene family, which includes KRAS (Kirsten rat sarcoma viral oncogene homologue), NRAS (neuroblastoma RAS viral oncogene homologue), and HRAS (Harvey rat sarcoma viral oncogene homologue), encodes small monomeric GTPases [7, 8]. In healthy cells, wild‐type RAS cycles between GDP‐bound (inactive) and GTP‐bound (active) states and plays a key role in regulating cell growth and survival [8, 9]. Hotspot mutations in codons 12, 13, or 61 of RAS genes are commonly found in tumours and VAs [10]. Mutant RAS genes are persistently in the GTP‐bound active state driving excessive mitogen‐activated protein kinase (MAPK) and phosphoinositide 3‐kinase (PI3K) signalling, unchecked cell proliferation, and increased survival [11].

Mutant RAS proteins were previously considered ‘undruggable’ due to their high affinity for GTP over GDP and lack of deep binding pockets for small molecule inhibitors [12, 13]. However, recently two main types of RAS inhibitors have developed: (1) RAS (ON) inhibitors, which target RAS in its active GTP‐bound state, and (2) RAS (OFF) inhibitors targeting the inactive GDP‐bound form. These new inhibitors have shown encouraging results in RAS‐driven cancers such as non‐small cell lung cancer (*KRAS* ^G12C^), colorectal cancer, and pancreatic ductal adenocarcinoma, raising the possibility of effectively targeting KRAS in KRAS‐driven tumours [12, 14, 15, 16, 17, 18, 19].

RMC‐7977 is a RAS (ON) multi‐selective inhibitor that directly targets active RAS isoforms (KRAS, HRAS, and NRAS) and is the paralog preclinical counterpart to the clinical drug candidate daraxonrasib [14, 20]. RMC‐7977 forms a binary complex with cyclophilin A and then a tri‐complex with the RAS‐GTP bound state, thereby suppressing downstream MAPK and PI3K signalling pathways by steric occlusion [12, 14, 20]. Despite its robust anti‐tumour efficacy in preclinical RAS‐driven cancer models [12, 14, 15, 16, 17, 18, 19], RMC‐7977 has not yet been evaluated in VMs with RAS mutations [4, 21, 22, 23, 24].

Patients with VMs, including brain, extracranial, and spinal arteriovenous malformations (AVMs), central conducting lymphatic anomalies (CCLA), Gorham‐Stout disease (GSD), and kaposiform lymphangiomatosis (KLA), frequently harbour activating mutations in the RAS/RAF/MEK cascade, such as KRAS, NRAS, and HRAS [4, 25, 26, 27]. Given its ability to target active GTP‐bound RAS, RMC‐7977 has the potential to block RAS‐driven VMs. This makes RMC‐7977 a potentially promising candidate for targeted therapy of VMs caused by mutant RAS.

RAS mutations have been identified in KLA patients, including *NRAS* p.Q61R (*NRAS* ^Q61R^) [4, 21, 22, 28], *KRAS* p.G12D [4, 22] (*KRAS* ^G12D^), and a novel *HRAS* mutation [4, 22] (E63_S65delinsDIPP, A59_Q61delinsGGSIL) [4, 22]. In addition, PI3KCA mutations (E545K and H1047R) and CBL (*CBL* ^Y774*^) mutation have also been identified in patients with KLA [22, 29]. Among these mutations, NRAS^Q61R^ is the most frequently identified to date, occurring in approximately 50%–90% of patients with KLA [21, 22]. The NRAS^Q61R^ variant is also a well‐established oncogenic driver in melanoma and several other malignancies [10, 12]. Patients with KLA have high levels of Angiopoietin‐2 (ANG‐2) in their blood [30, 31, 32] and *NRAS* ^Q61R^ expression in ECs can drive elevated expression of ANG‐2 [33, 34]. The mTORC1 inhibitor sirolimus remains the standard treatment for patients with KLA; however its efficacy is often limited to a partial response and/or non‐curative responses, underscoring the need for more effective treatment options [35]. Targeting mutant RAS directly is now possible with the development of the new generation of RAS‐targeted inhibitors [13, 14, 15, 19, 20, 36].

Given the availability of a preclinical RAS (ON) multi‐selective inhibitor, we used a human ECs model expressing *NRAS* ^Q61R^ to assess the therapeutic potential of RMC‐7977. The objectives of this study were: (1) To assess the in vitro efficacy of RMC‐7977 in ECs expressing *NRAS* ^Q61R^; (2) To evaluate whether RMC‐7977 can suppress *NRAS* ^Q61R^‐driven vessel overgrowth using an in vitro 3D angiogenesis assay; (3) To evaluate the in vivo efficacy of RMC‐7977 in suppressing NRAS^Q61R^‐driven vascular overgrowth using a mouse xenograft model.

## 2 Materials and Methods

Information on all key reagents and kits is provided in Tables S1 and S2.

### 2.1 Dose Response, Prevention, and Time‐Course Cell Culture Experiments

To study human ECs harbouring the NRAS^Q61R^ mutation, we used previously generated endothelial progenitor cells (EPCs) transduced with lentiviral vectors pCW‐*NRAS* ^*WT*^ or pCW‐*NRAS* ^Q61R^ [24, 33, 34]. Doxycycline (Dox; 2 μg/mL) was added to endothelial cell growth medium‐2 (EGM‐2; EBM‐2 supplemented with 20% fetal bovine serum [FBS] and complete growth factor supplements) to induce *NRAS* ^WT^ or *NRAS* ^Q61R^ expression in the EPCs [24, 33, 34]. A dose response experiment was performed to determine the most effective dose of RMC‐7977 to inhibit aberrant ECs features induced by *NRAS* ^Q61R^. Cells were plated at a seeding density of 100,000 cells per well in a 6‐well plate and allowed to attach for 24 h [33]. The next day cells were treated with No Dox (−), Dox (+), or Dox (+) in combination with RMC‐7977 (3.12, 6.25, 12.5, 25, 50 or 100 nM) or vehicle (control) for 72 h. For prevention studies RMC‐7977 was given at the same time as the cells were induced to express *NRAS* ^Q61R^ with Dox [33]. Cells were treated with vehicle control No Dox (−), Dox (+), or Dox (+) in combination with RMC (3.12, 12.5, or 50 nM) for 72 h. Images, cell culture media, and cell lysates were collected [33]. Cell morphology, ANG‐2 levels, and cell signalling were analysed for both experiments [33]. To determine the timing of RMC‐7977 inhibition of RAS‐activated RAS/RAF/MEK and PI3K/AKT/mTOR signalling pathways, a time‐course analysis of phosphorylated ERK (p‐ERK), phosphorylated S6K (p‐S6K), and phosphorylated AKT (p‐AKT) were performed on *NRAS* ^Q61R^ EPCs [33]. *NRAS* ^Q61R^ EPCs were plated at a seeding density of 50,000 cells per well in a 6‐well plate and allowed to attach overnight [33]. Cells were induced with Dox for 48 h to activate NRAS^Q61R^ expression. The culture medium was then replaced with fresh medium containing no Dox (−), Dox (+), or Dox (+) supplemented with RMC‐7977 (3.12, 12.5, or 50 nM) or vehicle control. Cell lysates were collected at 0.5, 1, 3, 6, 24, 48, and 72 h post treatment to assess the onset and duration of p‐ERK, p‐S6K, and p‐AKT inhibition by RMC‐7977 [33].

### 2.2 Western Blot Analysis

EPCs lysates were prepared from cells treated with No Dox (−), Dox (+), or Dox (+) in combination with RMC‐7977 (3.12–100 nM) and western blot analysis performed [33, 34]. Primary antibodies that detect phospho‐ERK (p‐ERK), total ERK, phospho‐S6K (p‐S6K), total S6K, phospho‐AKT (p‐AKT), total AKT, and ANG‐2 were incubated with the blot membranes in 5% Bovine Serum Albumin (BSA) overnight at 4°C or for 1 h at room temperature [33].

Primary antibodies specific for NRAS^Q61R^ (but not wild‐type NRAS) and actin were incubated in 5% NFDM overnight at 4°C or for 1 h at room temperature [33]. Secondary antibodies, including anti‐rabbit, anti‐goat, and anti‐mouse antibodies, were subsequently applied [24, 33, 34]. Western blot bands were quantified using Multi Gauge Software. Equal‐sized regions of interest were used to measure the intensity of each band, and background intensity was measured for each blot. Background‐corrected band intensities were obtained by subtracting the background signal from the corresponding band intensity. Relative protein expression was determined by calculating the ratio of phosphorylated protein to total protein (e.g., p‐ERK/ERK, p‐S6K/S6K, and p‐AKT/AKT) or by normalizing protein levels to actin, as indicated. Western blot analysis was performed using pooled lysates (*n* = 1); therefore, the densitometric measurements are descriptive and should be interpreted qualitatively.

### 2.3 Cell Proliferation and Migration Assays

To identify the optimal dose of RMC‐7977, we performed dose response experiments evaluating its effects on cell proliferation and migration [33]. For proliferation assays, NRAS^Q61R^ and NRAS^WT^ EPCs were seeded in 96‐well plates at a density of 2000 cells per well and allowed to attach overnight in EBM‐2 culture medium supplemented with 20% FBS, puromycin (0.5 mg/mL), Hoechst dye (two drops per 40 mL), and RMC‐7977 [33]. Treatment groups included: (1) No Dox with vehicle control, (2) Dox with vehicle control, or (3) Dox with RMC‐7977 (50 nM) treatment. Cell proliferation was monitored as described previously [33]. To assess cell migration, studies used either transwell inserts or scratch‐wound assays to create a defined gap into which cells could migrate [33]. For the transwell insert studies, NRAS^Q61R^ EPCs were seeded at a density of 10,000 cells per insert in 24‐well ibiTreated plates, with 4–5 replicates per treatment group [33]. Cells were treated with (1) No Dox with vehicle control, (2) Dox with vehicle control, and (3) Dox with RMC‐7977 (25 nM) (Figures S2 and S3). For scratch‐wound assays, NRAS^Q61R^ EPCs were seeded in 24‐well plates at a density of 15,000 cells per well. The treatment groups included: (1) No Dox with vehicle control, (2) Dox with vehicle control, and (3) Dox with RMC‐7977 (50 nM). Cells incubated for 48 h, then the media was removed, and a scratch created using a 200 μL pipette tip to generate a cell‐free gap for cell migration. Fresh media containing the corresponding treatments and hydroxyurea (Hu; 1 mM) was added to inhibit cell proliferation. Gap closure was monitored and imaged over 10 h using an EVOS M7000 Live‐Cell Imaging System (Invitrogen, Thermo Fisher Scientific) [33]. Images were analysed using ImageJ to quantify the percentage of gap closure over time [33].

### 2.4 In Vitro 3D Angiogenesis Assay

A 3D angiogenesis assay was performed (See schematic in Figure 4A) to evaluate the ability of RMC‐7977 to suppress the abnormal angiogenesis driven by NRAS^Q61R^ [24, 37]. Cytodex 3 microcarrier beads were incubated with *NRAS* ^Q61R^ EPCs at a concentration of 400 cells/bead for 4 h at 37°C [24, 37]. The next day, the beads coated with EPCs were resuspended in 2 mg/mL of fibrinogen, in a solution that contained 0.08 U/mL of aprotinin at a concentration of 500 beads/mL. Thrombin (0.75 U) and 0.5 mL beads/fibrinogen suspension were added per well of a 24‐well plate and incubated at 37°C for 20 min to allow fibrin clotting. Then, the gels were covered with human dermal fibroblasts at 30,000 cells/well and cultured in EBM2 media supplemented with 20% FBS and aprotinin (Figure S8). After 72 h the cell culture media was replaced with new media for 72 h as follows: (1) No Dox with vehicle control, (2) Dox with vehicle control, and (3) Dox with RMC‐7977 (12.5, 25, or 50 nM). The media was replaced every other day. Images were captured at different time points using EVOS M‐7000 live‐cell imaging system (Invitrogen by Thermo Fisher Scientific). Images were taken using 4 by 4 tile scan (16 scans total). Stitched tiles were used to quantify and analyse vascular area at day 1 and 14 of treatment (Figure S9). ImageJ was used to analyse the vascular area at day 1 and 14 of RMC‐7977 treatment.

### 2.5 Mouse Xenograft Model

All animal experiments were conducted with approval from the Institutional Animal Care and Use Committee (IACUC) at Cincinnati Children's Hospital Medical Center. The xenograft model used in this study has been previously described and published [24, 33]. *NRAS* ^Q61R^ EPCs were treated with No Dox or Dox (2 μg/mL) for 48 h in culture [24, 33]. Cells were trypsinized to detach them from the culture dish. EPCs (2.5 × 10^6^) were suspended in 200 μL of Matrigel (Corning) and subcutaneously injected into both flanks of 6–7‐week‐old male athymic NU/J mice (The Jackson Laboratory) on either a standard No Dox diet or a Dox‐containing diet (Day 0) [24, 33]. Two treatment protocols were used to evaluate the therapeutic effect of RMC‐7977 on growth of *NRAS* ^Q61R^ EPCs in vivo: (1) Cells were injected and after waiting 5 days for lesions to form (day 0–day 5) RMC‐7977 treatment (20 mg/kg) was administered by oral gavage for 8 days (Figure S13); (2) cells were injected and RMC‐7977 (25 mg/kg) treatment was started the next day by oral gavage and treated daily for 11 days (Figure 5A). Study groups included: (1) mice maintained on a No Dox diet and treated with vehicle control; (2) mice maintained on a Dox‐containing diet and treated with vehicle control; and (3) mice maintained on a Dox‐containing diet and treated with RMC‐7977. To prepare RMC‐7977 (25 mg/kg), RMC‐7977 (100 mg) was prepared at a concentration of 3.12 mg/mL. RMC‐7977 was initially dissolved in 1.6 mL of DMSO (1.6 mL; 5%), then diluted with a mixture of PEG300 (14.4 mL; 45%), Tween 80 (1.6 mL; 5%), and saline (14.4 mL; 45%). One day after Matrigel/cell injection, RMC‐7977 (25 mg/kg) or vehicle [control group; DMSO: 1.6 mL (5%), PEG300: 14.4 mL (45%), Tween 80: 1.6 mL (5%), Saline: 14.4 mL (45%)] was administered to mice once daily by oral gavage for 11 consecutive days. Mice were euthanized using CO_2_ and cervical dislocation. Xenograft plugs were removed by dissection, weighed, imaged using a Leica Stereo Microscope (M205 FA) and subsequently processed for histological analysis [24, 33].

### 2.6 Histological Analysis and Immunohistochemistry Staining

Paraffin‐embedded xenograft plugs were sectioned at 5 μm thickness onto glass slides and stained with haematoxylin and eosin (H&E) [33]. H&E‐stained xenograft plug sections were imaged using 2 × 3 tile scans to capture the entire section area (6 scans total) using a Lionheart Cell Imaging System (Agilent) [33]. Total and percentage vascular area (% of total area of plug) were measured using ImageJ software [33]. Immunohistochemistry (IHC) staining was performed using ulex europaeus agglutinin I (UEA‐1) to identify human ECs [24, 33] and p‐ERK staining to assess inhibition of MAPK pathway signalling [33]. EDTA‐ and citrate‐based antigen retrieval methods were used for UEA‐1 and p‐ERK staining, respectively [24, 33].

### 2.7 Statistical Analysis

Data are presented as mean ± standard deviation (SD). Comparisons between multiple groups were performed using one‐way analysis of variance (ANOVA) followed by Tukey's multiple comparisons test. For experiments involving repeated measures or paired observations, two‐way ANOVA followed by Bonferroni's multiple comparisons test was used. Statistical analyses and graphical representations were generated using GraphPad Prism. A *p*‐value < 0.05 was considered statistically significant.

## 3 Results

### 3.1 RMC‐7977 Blocks *NRAS* ^Q61R^ Driven MAPK Signalling and Reverses NRAS^Q61R^ Induced Signalling In Vitro

All original Western blot raw images are shown in the Figures S15–S23.

Densitometric analysis of the Western blot bands is shown in the Figures S24–S28.

**FIGURE 1:**
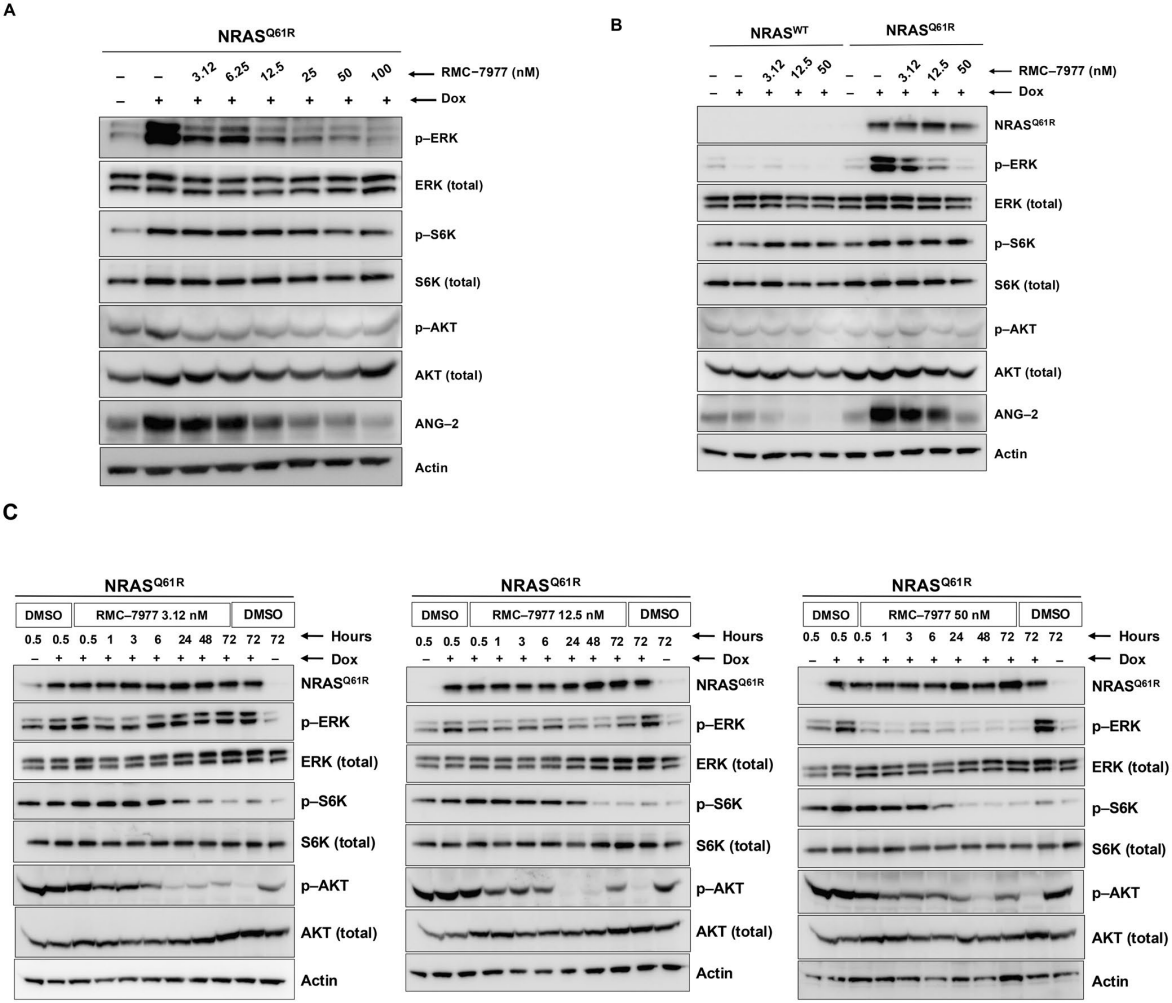
RMC‐7977 blocks *NRAS* ^Q61R^ activation of MAPK and reverses NRAS^Q61R^ induced signalling in vitro. (A) Western blot analysis of phospho‐ERK (p‐ERK), ERK (total), phospho‐S6K (p‐S6K), S6K (total), phospho‐AKT (p‐AKT), AKT (total), Angiopoietin‐2 (ANG‐2), and actin in cell lysates from cultured EPCs expressing NRAS^Q61R^ at 72 h. Treatments were no doxycycline (−), doxycycline (+) treatment, and doxycycline (+) in combination with RMC‐7977 (3.12, 6.25, 12.5, 25, 50, or 100 nM) added to the culture media for 72 h. Antibodies specific to phospho‐ERK (p‐ERK), ERK (total), phospho‐S6K (p‐S6K), S6K (total), phospho‐AKT (p‐AKT), AKT (total), Angiopoietin‐2 (ANG‐2), and actin were used. Representative blots of *n* = 3 independent replicate per treatment group. (B) Western blot analysis of NRAS^Q61R^, phospho‐ERK (p‐ERK), ERK (total), phospho‐S6K (p‐S6K), S6K (total), phospho‐AKT (p‐AKT), AKT (total), Angiopoietin‐2 (ANG‐2), and actin in cell lysates from cultured EPCs expressing NRAS^WT^ or NRAS^Q61R^. Treatment groups were no doxycycline (−), doxycycline (+), and doxycycline (+) in combination with RMC‐7977 (3.12, 12.5, or 50 nM) added to the culture media for 72 h. Antibodies specific to NRAS^Q61R^, phospho‐ERK (p‐ERK), ERK (total), phospho‐S6K (p‐S6K), S6K (total), phospho‐AKT (p‐AKT), AKT (total), Angiopoietin‐2 (ANG‐2), and actin were used. *n* = 6 independent replicate per treatment group. (C) Western blot analysis of phospho‐ERK (p‐ERK), phospho‐S6K (p‐S6K), and phospho‐AKT (p‐AKT) time‐course analysis in cell lysates from cultured EPCs expressing *NRAS* ^Q61R^. Cells were treated with doxycycline (+) for 48 h to induce *NRAS* ^Q61R^ expression. The media was replaced with new media containing no doxycycline (−), doxycycline (+), and doxycycline (+) in combination with RMC‐7977 (3.12, 12.5, or 50 nM) added to the cell culture media for 0.5, 1, 3, 6, 24, 48, and 72 h. Antibodies specific to NRAS^Q61R^, phospho‐ERK (p‐ERK), ERK (total), phospho‐S6K (p‐S6K), S6K (total), phospho‐AKT (p‐AKT), AKT (total), and actin were used. *n* = 3 independent replicate per treatment group.

In the dose response experiment, western blot analysis of EPCs lysates showed that ERK and S6K phosphorylation increased with Dox‐induced *NRAS* ^Q61R^ expression compared to No Dox controls (Figure 1A and Figures S15, S16 and S24). Lower concentrations of RMC‐7977 (3.12 and 6.25 nM) did not prevent the increases in ERK phosphorylation in NRAS^Q61R^ EPCs (Figure 1A and Figures S15, S16 and S24). In contrast, higher concentrations of RMC‐7977 (12.5–100 nM) effectively blocked the rise in p‐ERK levels in these cells (Figure 1A and Figures S15 and S24). Lower concentrations of RMC‐7977 (3.12–25 nM) did not reduce the increases in S6K phosphorylation in NRAS^Q61R^ EPCs (Figure 1A and Figures S16 and S24). However, higher concentrations of RMC‐7977 (50–100 nM) reduced the increases in p‐S6K slightly (Figure 1A and Figures S16 and S24). AKT phosphorylation increased with Dox‐induced *NRAS* ^Q61R^ expression compared to No Dox controls (Figure 1A and Figures S17 and S24). ANG‐2 levels, elevated in Dox‐induced *NRAS* ^Q61R^ EPCs, were reduced by a wide range of RMC‐7977 doses both in cell lysates and culture media (12.5–100 nM) (Figure 1A, Figure 3C, and Figures S18 and S24). In the prevention study, Dox‐induced *NRAS* ^Q61R^ EPCs had elevated levels of p‐ERK and ANG‐2 (Figure 1B and Figures S19 and S25). Treatment with RMC‐7977 (50 nM) blocked increased levels of p‐ERK and ANG‐2 at 72 h (Figure 1B and Figures S19 and S25). RMC‐7977 treatment (3.12, 12.5, 50 nM) slightly reduced basal levels of p‐ERK and ANG‐2 in NRAS^WT^ EPCs (Figure 1B and Figures S19 and S25). Dox‐induced *NRAS* ^Q61R^ EPCs had elevated levels of p‐S6K compared to no Dox‐treated cells (Figure 1B and Figures S19 and S25). RMC‐7977 treatments did not reduce the increase in p‐S6K (Figure 1B and Figures S19 and S25). p‐AKT did not increase with Dox‐induced *NRAS* ^Q61R^ expression compared to no Dox controls (Figure 1B and Figures S20 and S25). In the western blot analysis of the time course 3.12 nM RMC‐7977 caused only transient reductions in ERK phosphorylation in NRAS^Q61R^ EPCs (Figure 1C and Figures S21 and S26), whereas higher doses of RMC‐7977 (12.5 and 50 nM) reduced p‐ERK levels at all time points (Figure 1C and Figures S22, S23 and S27, S28). Interestingly, p‐S6K and p‐AKT were reduced over time in all RMC‐7977 treatments 3.12 nM, 12.5 nM, and 50 nM (Figure 1C and Figures S22, S23 and S27, S28). Whether this is due to degradation or inactivation of the pathway remains unclear, and future studies are needed to address this question.

### 3.2 RMC‐7977 Blocks *NRAS* ^Q61R^ Induced Cell Proliferation and Migration

**FIGURE 2:**
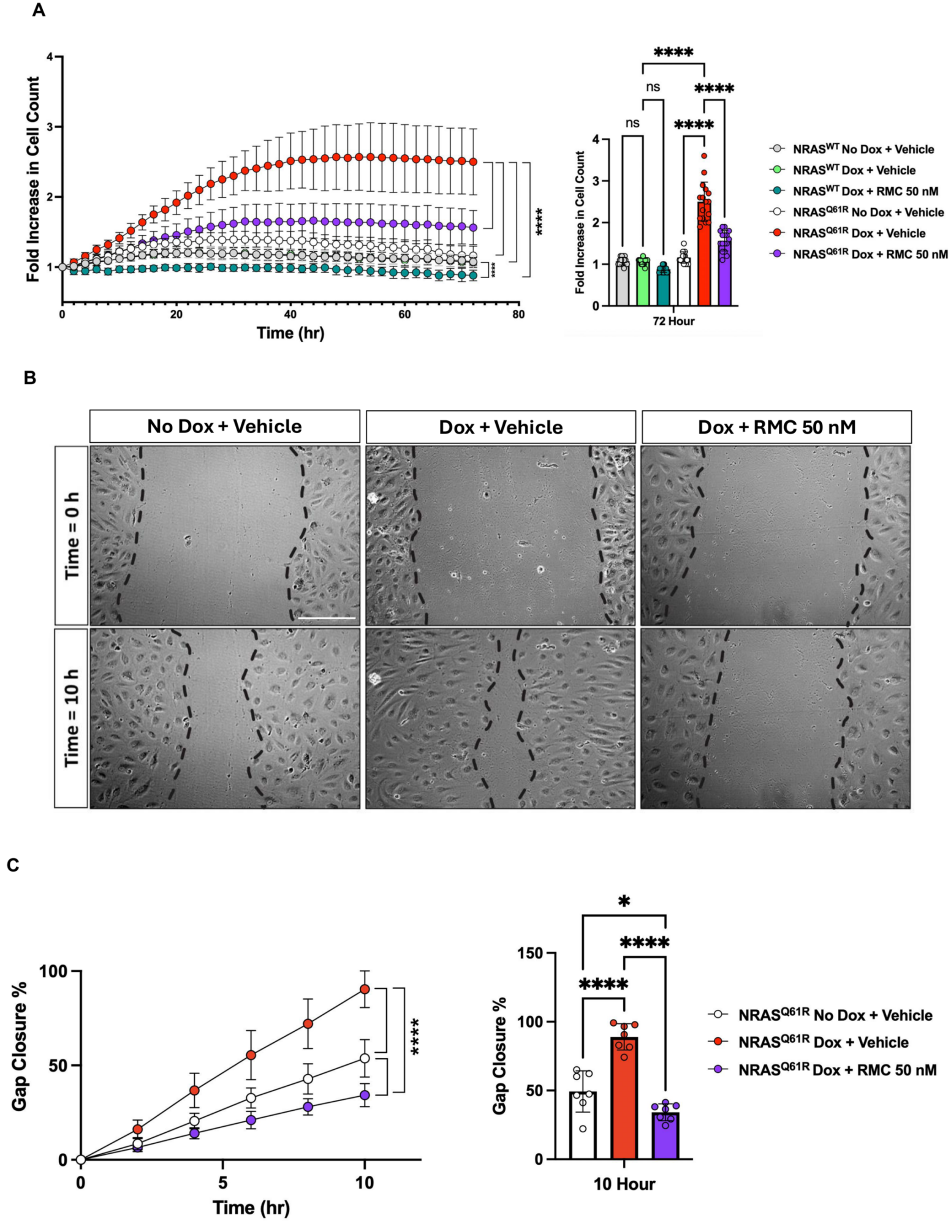
RMC‐7977 Blocks *NRAS* ^Q61R^ Induced Cell Proliferation and Migration. (A) Proliferation of EPCs expressing *NRAS* ^WT^ and *NRAS* ^Q61R^ was measured using a Lionheart automated microscope system every 2 h for 72 h. EPCs treatment groups included no doxycycline and vehicle (No Dox + Vehicle), doxycycline and vehicle (Dox + Vehicle), and doxycycline with RMC‐7977 (Dox + RMC 50 nM) added to the cell culture media. Data points are mean ± SD; *n* = 16 wells per treatment. Two‐way analysis of variance (ANOVA) followed by Bonferroni test for multiple comparisons. ns, not significant and ****p < 0.0001 for times ≥ 72 h. Histogram shows proliferation at 72 h across the different treatment groups. One‐way analysis of variance (ANOVA) followed by Tukey's multiple comparison test. ns, not significant, *p < 0.0107, and ****p < 0.0001. (B) Representative images of migration of EPCs expressing *NRAS* ^Q61R^ at time = 0 and time = 10 h after the gap was created. EPCs groups included no doxycycline and vehicle (No Dox + Vehicle), doxycycline and vehicle (Dox + Vehicle), doxycycline (Dox) with RMC‐7977 (50 nM) added to the cell culture media for 48 h. Representative images are shown, *n* = 3–6 wells analysed. Scale bar = 275 μm. (C) Quantitation of migration of EPCs expressing *NRAS* ^Q61R^ measured every 2 h from 0 h to 10 h after creating the gap. Gap closure was measured using ImageJ and percentage closure calculated. EPCs groups included no doxycycline and vehicle (No Dox + Vehicle), doxycycline and vehicle (Dox + Vehicle), and treatment with doxycycline and RMC‐7977 (Dox + RMC 50 nM). Data points are mean ± SD; *n* = 6–7 wells per treatment. Two‐way analysis of variance (ANOVA) followed by Bonferroni test for multiple comparisons. ****p < 0.0001. Histogram shows gap closure at 10 h for the study groups. One‐way ANOVA followed by Tukey's multiple comparison test. *p < 0.0462 and ****p < 0.0001.

RMC‐7977 (50 nM) treatment reduced basal cell proliferation of NRAS^WT^ EPCs over time, but it was not significant at 72 h (Figure 2A). Cell proliferation increased with Dox‐induced *NRAS* ^Q61R^ expression compared to No Dox controls (Figure 2A and Figure S1). 12.5 nM RMC‐7977 did not reduce cell proliferation at 72 h (Figure S1). However, higher doses of RMC‐7977 (25 nM and 50 nM) treatment reduced NRAS^Q61R^ EPCs proliferation at 72 h (Figure 2A and Figure S1). The cell migration rate was higher in Dox‐induced *NRAS* ^Q61R^ EPCs compared to No Dox controls (Figure 2B,C and Figures S2, S3). While 25 nM RMC‐7977 did not reduce the rate of gap closure at 10 h (Figures S2, S3), 50 nM RMC‐7977 suppressed the migration rate of NRAS^Q61R^ EPCs at 10 h (Figure 2B,C).

### 3.3 RMC‐7977 Blocks *NRAS* ^Q61R^ Driven Abnormal Cell Morphology and Increased ANG‐2 Production

**FIGURE 3:**
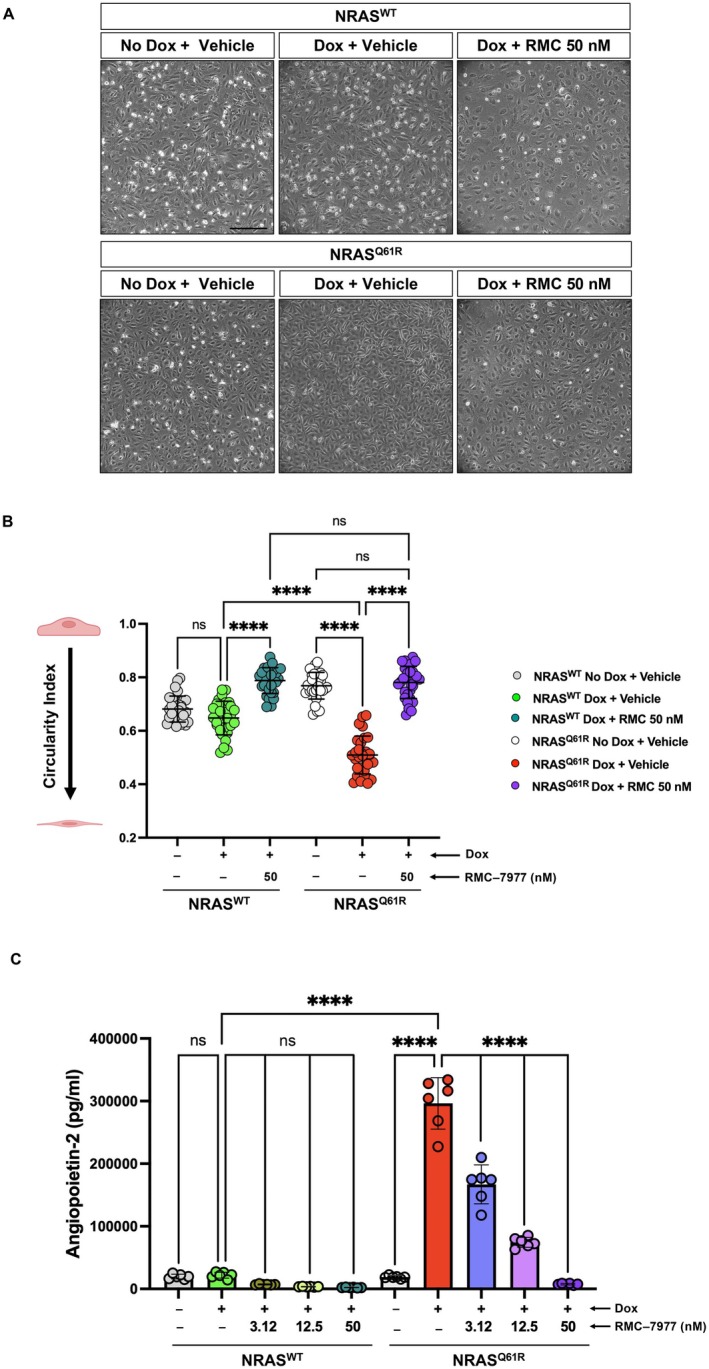
RMC‐7977 blocks *NRAS* ^Q61R^ ‐induced abnormal cell morphology. (A) Representative images of *NRAS* ^WT^ and *NRAS* ^Q61R^ EPCs at 72 h. EPCs treatment groups included no doxycycline and vehicle (No Dox + Vehicle), doxycycline and vehicle (Dox + Vehicle), and doxycycline (Dox) with RMC‐7977 (50 nM) added to the cell culture media. Representative images are shown, *n* = 6 wells analysed. Scale bar = 400 μm. (B) Cell circularity index of EPCs expressing *NRAS* ^WT^ and *NRAS* ^Q61R^ measured at 72 h. EPCs treatment groups included no doxycycline and vehicle (−), doxycycline and vehicle (+), and doxycycline (+) with RMC‐7977 (50 nM) added to the cell culture media. Data points are mean ± SD. *n* = 6 wells per group. One‐way ANOVA was performed with Tukey's multiple comparison test. ns, not significant and ****p < 0.0.0001. (C) Angiopoietin‐2 in the cell culture medium of EPCs expressing *NRAS* ^WT^ and *NRAS* ^Q61R^ measured using an ELISA. EPCs treatment groups included no doxycycline and vehicle (−) and doxycycline and vehicle (+), and doxycycline with different doses of RMC‐7977 (3.12, 12.5, or 50 nM) added to the cell culture media for 72 h. Data points are mean ± SD. *n* = 6 wells per group. One‐way ANOVA was performed with Tukey's multiple comparison test. ns, not significant and ****p < 0.0001.

EPCs expressing NRAS^WT^ treated with doxycycline and RMC‐7977 (50 nM) were significantly more rounded compared to NRAS^WT^ treated with doxycycline and vehicle (Figure 3A,B and Figure S6). As previously reported [24, 33, 34] doxycycline‐induced NRAS^Q61R^ expression caused a shift in cell morphology from the typical endothelial cobblestone shape to an elongated and spindle‐like phenotype (Figure 3A,B and Figures S4–S6). RMC‐7977 (50 nM) treatment prevented this switch in morphology as the circularity index was not different from No Dox control (Figure 3B and Figure S6). For the dose response experiment, RMC‐7977 treatment (3.12–100 nM) prevented the shift to a spindle‐shaped morphology in a dose dependent manner (Figures S4, S5). EPC expressing NRAS^WT^ treated with RMC‐7977 (3.12, 12.5, 50 nM) did not significantly reduce ANG‐2 levels in the cell culture medium at 72 h (Figure 3C). ANG‐2 levels were higher in the cell culture media of NRAS^Q61R^ EPCs compared to No Dox NRAS^Q61R^ and NRAS^WT^ EPCs (Figure 3C and Figure S6). RMC‐7977 treatment (3.12, 12.5, 50 nM) reduced ANG‐2 levels of Dox‐induced NRAS^Q61R^ EPCs (by 44%, 75%, and 97% respectively) (Figure 3C). For the dose response experiment, RMC‐7977 treatment (3.12–100 nM) reduced ANG‐2 levels in the cell culture media in a dose dependent manner (37%–92%) (Figure S7).

### 3.4 RMC‐7977 Blocks Overgrowth of *NRAS* ^Q61R^ ECs in a 3D Angiogenesis Assay

**FIGURE 4:**
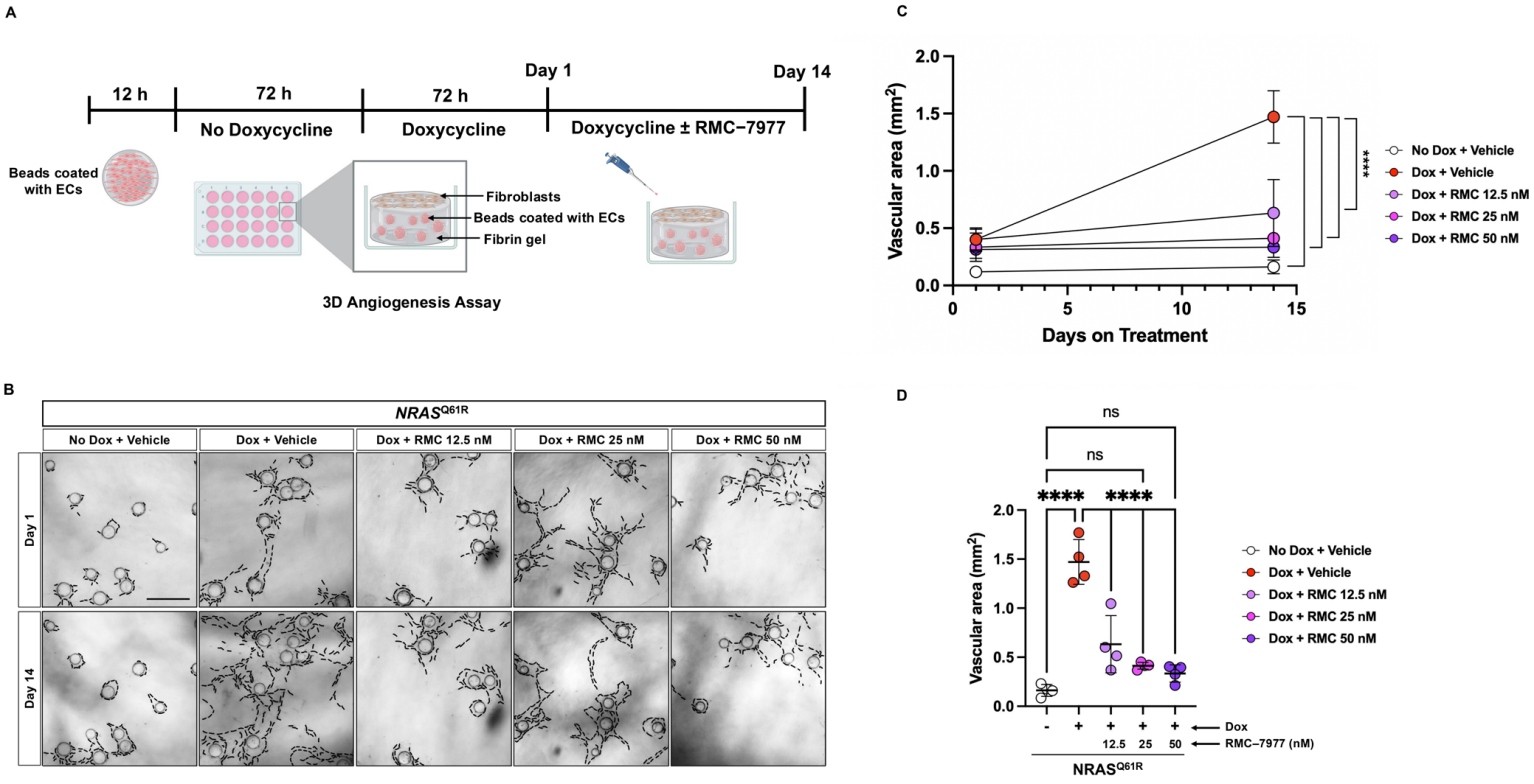
RMC‐7977 Blocks Overgrowth of *NRAS* ^Q61R^ ECs in a 3D Angiogenesis Model. (A) Experimental protocol and schematic of 3D angiogenesis model. Beads coated with endothelial cells and embedded in fibrin gel and coated with fibroblast cells. Cells were allowed to grow without doxycycline for 72 h. After 72 h, doxycycline was added to the cell culture media for 72 h. After 72 h media was removed and replaced with new media containing no doxycycline and vehicle (No Dox + Vehicle), doxycycline and vehicle (Dox + Vehicle), or doxycycline (+) in combination with RMC‐7977 (12.5, 25, or 50 nM). Images were taken on day 1 and day14 of treatment. (B) Representative images of vascular structures formed by *NRAS* ^Q61R^ EPCs between and around the beads on day 1 and day 14. EPCs treatment groups included no doxycycline and vehicle (No Dox + Vehicle), doxycycline and vehicle (Dox + Vehicle), and doxycycline (Dox) in combination with RMC‐7977 (12.5, 25, or 50 nM). Representative images are shown, *n* = 4 wells analysed. Scale bar = 650 μm. (C) Quantification of vascular area formed by EPCs expressing *NRAS* ^Q61R^ measured on day 1 and day 14. EPCs treatment groups included no doxycycline and vehicle (No Dox + Vehicle), doxycycline and vehicle (Dox + Vehicle), and doxycycline (Dox) in combination with RMC‐7977 (12.5, 25, or 50 nM). *n* = 4 wells analysed. Two‐way ANOVA was performed with Bonferroni test for multiple comparisons. ****p < 0.0.0001. (D) Quantification of Vascular area formed by EPCs expressing *NRAS* ^Q61R^ measured at day 14. EPCs treatment groups include no doxycycline and vehicle (No Dox + Vehicle), doxycycline and vehicle (Dox + Vehicle), and doxycycline (Dox) in combination with RMC‐7977 (12.5, 25, or 50 nM). *n* = 4 wells analysed. One‐way ANOVA was performed with Tukey's multiple comparison test. ns, not significant, **p < 0.0067, and ****p < 0.0.0001.

Figure S8 shows the beginning of vascular structure formation at 72 h for no Dox and Dox‐treated cells on beads, prior to treatment with RMC‐7977. Dox‐induced *NRAS* ^Q61R^ EPCs formed enlarged disorganized structures with higher vascular area than no dox controls (Figure 4B–D and Figure S9). RMC‐7977 treatment (12.5, 25, and 50 nM) blocked the formation of enlarged disorganized structures and reduced vascular area by 57%, 72%, 77%, respectively (Figure 4B–D).

### 3.5 RMC‐7977 Treatment Reduces *NRAS* ^Q61R^ Induced Vascular Overgrowth and p‐ERK In Vivo

**FIGURE 5:**
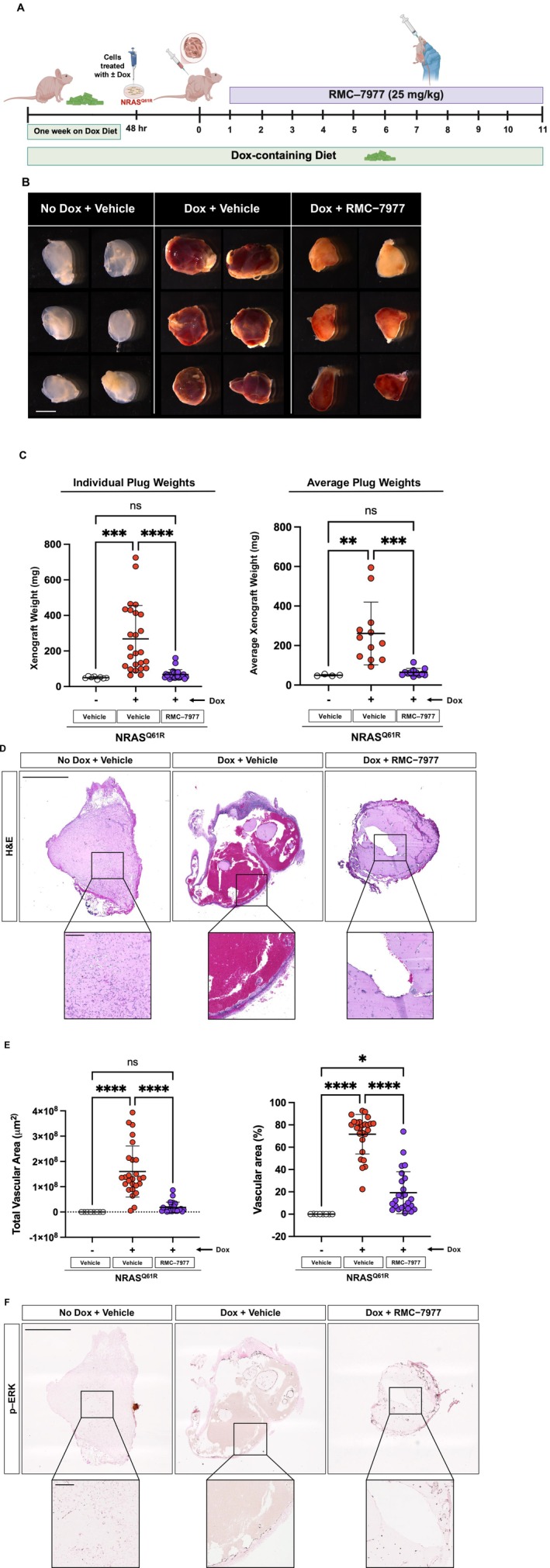
Effect of RMC‐7977 on *NRAS* ^Q61R^ Induced Vascular Overgrowth. (A) Schematic of the xenograft model protocol: Experimental timeline showing doxycycline (Dox) administration, the time of cell injection day (0), treatment period with RMC‐7977 or vehicle (day 1–11), and the time point for xenograft plug harvest (day 11). (B) Representative images of xenograft plugs on day 11. Treatment groups included no doxycycline and vehicle (No Dox + Vehicle), doxycycline and vehicle (Dox + Vehicle), and doxycycline in combination with RMC‐7977 treatment (Dox + RMC‐7977). Scale bar = 4 mm. (C) Graphs of xenograft plug weights on day 11. Data points are individual plugs (left) and average plugs weight (right) with 2 plugs per mouse: 4 mice received no doxycycline (No Dox + Vehicle), 13 mice on doxycycline‐containing diet (Dox + Vehicle), and 13 mice doxycycline diet and treated by oral gavage with RMC‐7977 (25 mg/kg; Dox + RMC‐7977). One‐way ANOVA followed by Tukey's multiple comparisons test for individual plugs. ns, not significant, ***p < 0.0002, ****p < 0.0001. Averaged plugs weight, ns, not significant, **p < 0.0055, ***p < 0.0004. (D) Representative histology of xenograft plugs harvested on day 11. Treatment groups included no doxycycline and vehicle (No Dox + Vehicle), doxycycline and vehicle (Dox + Vehicle), and doxycycline with RMC‐7977 (25 mg/kg; Dox + RMC‐7977). Tissue sections were stained with haematoxylin and eosin (H&E). Images were acquired using a Lionheart Imaging System. 4× scale bar = 2000 μm; 10× scale bar = 300 μm. (E) Total vascular area and vascular area percentage (vascular area normalized to total plug area) in H&E sections was measured using ImageJ. Graphs were generated using Prism 10. Treatment groups included no doxycycline and vehicle (No Dox + Vehicle), doxycycline and vehicle (Dox + Vehicle), and doxycycline with RMC‐7977 treatment (25 mg/kg; Dox + RMC‐7977). One‐way ANOVA with Tukey's multiple comparisons test. For total vascular area: Ns = not significant and ****p < 0.0001. For vascular area percentage: *p < 0.0205 and ****p < 0.0001. (F) Immunohistochemistry staining of xenograft plug sections for phospho‐ERK (p‐ERK). Images were acquired using Lionheart Imaging system. Representative images are shown for each treatment group, no doxycycline and vehicle (No Dox + Vehicle), doxycycline and vehicle (Dox + Vehicle), and doxycycline with RMC‐7977 treatment (25 mg/kg; Dox + RMC‐7977). 4× Scale bar = 2000 μm, 10× Scale bar = 300 μm.

In the in vivo xenograft mouse model (Figure S13), xenograft plug weight from *NRAS* ^Q61R^ EPCs in Dox‐treated mice were higher compared to No‐Dox controls (Figure S14). In the first treatment protocol, RMC‐7977 treatment (20 mg/kg) was initiated 5 days after cell injection and daily for 8 days. However, this treatment protocol did not significantly reduce xenograft plug weights compared to Dox‐treated cells (Figure S14). Therefore, a second treatment protocol was used with a higher dose of RMC (25 mg/kg) and treatment initiated 1 day after cell injection and continued daily for 11 days (Figure 5A). In the in vivo xenograft mouse model (Figure 5A), xenograft plugs from *NRAS* ^Q61R^ EPCs in Dox‐treated mice were enlarged and visibly bloody compared to No‐Dox controls (Figure 5B and Figure S10). In contrast, plugs from mice treated with RMC‐7977 were generally smaller and less bloody (Figure 5B and Figure S10). Weights of *NRAS* ^Q61R^ EPCs xenograft plugs of Dox‐treated mice were 5.3‐fold higher than those in the No Dox group (Figure 5C). In contrast, xenograft plugs from mice treated with RMC‐7977 weighed 75% less. Histology revealed that the *NRAS* ^Q61R^ xenograft plugs from Dox‐treated mice contained markedly enlarged vessels, whereas plugs from mice treated with RMC‐7977 contained much smaller vessels (Figure 5D). Total Vascular area and percentage vascular area in sections of xenograft plugs from *NRAS* ^Q61R^ EPCs on Dox were higher than No Dox controls (Figure 5D,E). In contrast, in mice treated with RMC‐7977 there was an 89% reduction in total vascular area and 73% reduction in percent vascular area (vascular area was expressed as a percentage of total area) (Figure 5D,E). IHC staining showed that p‐ERK levels were higher in ECs lining the vessels in the Dox‐induced NRAS^Q61R^ plugs compared to No Dox controls, whereas p‐ERK staining was reduced in plugs from mice treated with RMC‐7977 (Figure 5F). These results demonstrate that RMC‐7977 effectively inhibits MAPK pathway activation in NRAS^Q61R^ xenografts. Staining of xenograft plug sections with UEA‐I showed that the vessels in the xenografts were human ECs rather than mouse ECs (Figure S11). Mouse body weights did not differ between the treatment groups over the course of the study, suggesting the treatment was generally well tolerated, consistent with previous reports (Figure S12) [14].

## 4 Discussion

This study evaluates the effectiveness of RAS (ON) multi‐selective inhibitor RMC‐7977 in preclinical human endothelial cell models expressing the hyperactive NRAS^Q61R^ mutant protein. In vitro, RMC‐7977 suppressed *NRAS* ^Q61R^‐driven increases in ERK phosphorylation, cell proliferation, migration, spindling morphology, and elevated ANG‐2 production, with the degree of inhibition varying depending on the dose. RMC‐7977 also prevented the formation of enlarged disorganized vascular structures in an in vitro 3D angiogenesis assay using *NRAS* ^Q61R^ expressing ECs. In mouse xenograft studies, RMC‐7977 treatment lowered ERK phosphorylation and reduced overgrowth of vessels generated by NRAS^Q61R^ ECs, as evidenced by decreased plug weight and reduced vascular area. Hence, these in vitro and in vivo studies demonstrate that RMC‐7977 effectively inhibits RAS‐dependent signalling pathways, normalizes aberrant ECs phenotypes, and suppresses pathological vascular overgrowth driven by the NRAS^Q61R^ mutation.

Recent studies using RMC‐7977 demonstrated that oncogenic signalling depends more on the specific RAS mutation than on the RAS isoform or tumour type [38]. Tumours with RAS^Q61X^ mutations show constitutive activation of the MAPK pathway that is largely independent of receptor tyrosine kinase (RTK) signalling. However, PI3K/AKT/mTOR signalling remains primarily driven by RTKs and is therefore less impacted by direct RAS inhibition. In contrast, RAS^G12X^ mutants activate both MAPK and PI3K pathways [38]. As a result, inhibition of RAS^G12X^ mutants suppresses both MAPK and PI3K signalling, whereas inhibition of RAS^Q61X^ primarily reduces MAPK signalling with minimal effect on PI3K activity [38]. These mutation‐specific signalling differences may help explain our findings. In our model, NRAS^Q61R^ primarily drives MAPK signalling, and treatment with RMC‐7977 effectively reduced MAPK activation while having minimal effects on PI3K signalling. These findings suggest that MEK inhibitors or direct RAS inhibition with RMC‐7977 may represent effective therapeutic strategies for RAS^Q61X^‐driven vascular anomalies. Given the improved tolerability profile reported for RMC‐7977 [39], future studies could investigate whether combining RMC‐7977 with sirolimus may offer a more effective strategy for dual MAPK and mTOR pathway inhibition, potentially overcoming the toxicity that has limited previous combination approaches [40].

Sirolimus is a standard treatment for patients with vascular anomalies and KLA [41, 42]. However, in some patients with KLA sirolimus treatment was not effective at reducing pleural and pericardial effusions, thrombocytopenia, and ANG‐2 levels [29, 32, 43, 44]. Trametinib treatment showed better efficacy in these patients [29, 32, 43, 44]. Other case reports of CLAs reported that while trametinib or sirolimus treatment alone was sometimes not effective, using them in combination at low concentration can be very effective [45]. In addition, sirolimus treatment did not prevent the spindle‐shaped morphological changes but did attenuate ANG‐2 levels in EPCs expressing NRAS^Q61R^ [34]. However, trametinib treatment blocked the transition to a spindle‐shaped morphology and prevented the increase in ANG‐2 levels [33, 34]. Whether direct inhibition of mutant RAS signalling in KLA patients with a multi‐selective RAS inhibitor would be as or more effective than sirolimus or trametinib alone or in combination remains to be determined.

The mechanism of action of the RAS ‘ON’ inhibitor RMC‐7977 differs fundamentally from RAS “OFF” inhibitors such as KRAS^G12C^ inhibitors sotorasib and adagrasib. RMC‐7977 acts by forming a tri‐complex with a cyclophilin A and RAS‐GTP, in its bound state in a way that prevents downstream signalling. RMC‐7977 targets wild‐type and mutant RAS forms of NRAS, KRAS, and HRAS. This broad activity represents a limitation in our study, as RMC‐7977 is not an NRAS‐specific inhibitor and so may also affect the function of wild‐type RAS proteins. However, this feature may provide an advantage clinically given that other RAS mutations have been found in KLA [22]. A RAS inhibitor with broad specificity such as RMC‐7977 might also be useful in the treatment of patients for which the exact RAS mutation cannot be identified, and where other treatments have failed. Although RMC‐7977 targets both mutant and wild‐type RAS isoforms, it has more impact on activated mutant RAS, locked into GTP‐bound “ON” state, than wild‐type RAS which cycles between active GTP‐bound state (ON) and inactive GDP‐bound state (OFF) [14, 39].

Previous studies evaluated a RAS ‘OFF’ inhibitor—sotorasib, a KRAS^G12C^ inhibitor, in genetic mouse models and two patients with AVMs carrying the KRAS^G12C^ mutation [46]. In contrast, our study evaluates a RAS ‘ON’ inhibitor in preclinical human endothelial cell models of VMs driven by an NRAS mutation. We demonstrate that a RAS (ON) multi‐selective inhibitor can block NRAS^Q61R^‐driven endothelial dysfunction and pathological vascular overgrowth. These results provide a rationale for targeting mutant RAS signalling as a therapeutic strategy for patients with KLA. More broadly, these findings may have implications for other vascular anomalies caused by RAS mutations [4], supporting the development of precision medicine approaches for this underserved patient population in urgent need of new therapeutic options.

While this study provides important insights into NRAS^Q61R^‐driven endothelial pathology and therapeutic targeting, several limitations should be considered. First, the doxycycline‐inducible overexpression system may not fully recapitulate endogenous levels of NRAS^Q61R^ in patient lesions, where mutations are often mosaic and present at low allele frequencies [21, 22]. This limitation may influence both the degree of pathway activation and the response to therapeutic treatments. Second, ANG‐2 can be used as a diagnostic biomarker to distinguish KLA from other types of CLAs [30], although the exact pathway driving this in KLA patients remains unclear. Previous experimental studies have shown that NRAS^Q61R^ drives increases in ANG‐2 expression in vitro and in endothelial‐targeted NRAS^Q61R^ genetic mouse models [33, 34, 37, 47]. Treatment with trametinib (MEK inhibitor) blocked NRAS^Q61R^‐driven increases in ANG‐2 in vitro [33, 34]. In addition, ANG‐2 levels were elevated in a patient with KLA (without known genetic mutation) and decreased with trametinib treatment compared to sirolimus treatment [32]. Whether ANG‐2 contributes directly to KLA pathogenesis or vascular overgrowth remains unclear, and future studies will be required to determine if it has a mechanistic role, besides being a useful biomarker [30, 32]. Third, RMC‐7977 is a multi‐selective RAS (ON) inhibitor, but in this study, we only evaluated RMC‐7977 treatment in NRAS^WT^ and NRAS^Q61R^ ECs models. Patients with KLA can harbour different RAS mutations, including KRAS^G12D^ and HRAS mutations [22], therefore future studies could evaluate whether RMC‐7977 is effective at targeting different RAS mutations in vascular anomalies [4]. In our study, NRAS^WT^ ECs showed basal levels of p‐ERK signalling and cell proliferation, both of which were reduced following treatment with RMC‐7977. In previous studies both RMC‐7977 and RMC‐6236 have been reported to have limited effects on normal, healthy cells [20, 39]. Fourth, our in vitro studies utilised human EPCs, which may not fully reflect the behaviour of LECs with the NRAS^Q61R^ mutation. However, our previous work showed that LECs expressing NRAS^Q61R^ exhibit very similar pathological features to EPCs, including MAPK hyperactivation, increased proliferation and migration, elevated ANG‐2 production, and formation of enlarged vascular channels in an in vitro model [37]. Nevertheless, NRAS^Q61R^‐expressing LECs have not yet been evaluated for their response to MEK inhibitors or RMC‐7977. Importantly, previous studies using KRAS^G12D^‐expressing human LECs demonstrated that MEK inhibition by trametinib reduced cell proliferation, migration, spindle‐like morphology, and partially corrected dysregulated gene expression, supporting the importance of MAPK signalling in lymphatic endothelial models [48]. Together, these findings support the possibility that MEK inhibition or RAS inhibition may also be effective in LECs expressing NRAS^Q61R^. Future studies are needed to directly evaluate these therapeutic strategies in LEC models. Fifth, although the xenograft model is a valuable tool for evaluating therapeutic responses in vivo, it does not fully recapitulate the complex pathogenesis of KLA [33]. Treatment with the RAS inhibitor in this study reduced vascular growth in the xenografts and p‐ERK staining, demonstrating effective suppression of NRAS^Q61R^‐driven vascular overgrowth. However, this model lacks some of the key clinical features of KLA, including elevated ANG‐2 levels, splenomegaly, thrombocytopenia, pleural and pericardial effusions, and interactions with the native immune microenvironment. Sixth, in our study two RMC‐7977 treatment groups were used (20 and 25 mg/kg). However, RMC‐7977 treatment at 20 mg/kg did not significantly reduce xenograft plug weight. This could be due to two factors: (1) treatment was started 5 days after lesion formations had already been well established/formed and (2) the treatment duration was relatively short, only 8 days. To overcome these challenges, we changed our experimental design based on published cancer data on RMC‐7977 [13]. These studies used xenograft mouse models and started treating with RMC‐7977 (25 mg/kg) the same day of cell injections and treated mice daily via oral gavage for 16 days [13]. In these short‐term experiments, it was necessary to start treating after cell injections [13]. Therefore, we followed their approach. Seventh, our study primarily evaluated short‐term therapeutic responses and did not address long‐term efficacy, toxicity, or mechanisms of resistance. Therefore, in the future it will be important to extend our studies to address these issues and utilise other models of KLA as well [34, 47]. Furthermore, it will be important to determine whether there are any sex differences in the models as our xenograft studies only used male mice [34, 47, 49, 50, 51]. Finally, Western blot analyses were performed using pooled lysates (*n* = 1). Therefore, the observed changes in protein expression should be interpreted as descriptive rather than statistically validated quantitative findings.

In summary, this study demonstrates that NRAS^Q61R^ can be key driver of pathological endothelial cell behaviour and that targeting the RAS pathway represents a promising therapeutic strategy in patients with this mutation, such as KLA. Direct RAS inhibition effectively suppresses many disease‐associated phenotypes both in vitro and in vivo models. These findings advance our understanding of KLA pathogenesis and provide a foundation for the development of targeted therapies for RAS‐driven vascular anomalies. Continued investigation into downstream signalling mechanisms, combination therapeutic strategies, and clinically relevant animal models will be essential for improving outcomes for patients with this rare and aggressive disease.

## Author Contributions

**George S. Zaky:** writing – review and editing, software, formal analysis. **Patricia Pastura:** writing – review and editing, visualization. **Sara Alharbi:** conceptualization, writing – original draft, writing – review and editing, investigation, methodology, validation, visualization, formal analysis, software, data curation, project administration. **Andrew M. Waters:** writing – review and editing, supervision. **Svatava Merkle:** writing – review and editing, methodology. **Timothy D. Le Cras:** writing – review and editing, supervision, funding acquisition, project administration. **C. Griffin McDaniel:** writing – review and editing, methodology.

## Funding

This work was supported by Lymphatic Malformation Institute. National Institutes of Health HL156866.

## Disclosure

No artificial intelligence was used in developing any portion of the manuscript preparation, studies, or models.

## Conflicts of Interest

A. M. Waters has consulted for and receives non‐financial support from Revolution Medicines.

## Supporting information


**Figure S1:** RMC‐7977 dose‐response analysis of proliferation in EPCs expressing NRAS^Q61R^ Cell counts were measured using a Lionheart automated microscope system every 2 h for 72 h. EPCs groups included no doxycycline and vehicle (No Dox + Vehicle), doxycycline and vehicle (Dox + Vehicle), and doxycycline with RMC‐7977 (Dox + RMC 12.5, 25, or 50 nM) added to the cell culture media. Data points are mean ± SD; *n* = 16 wells per treatment. Two‐way analysis of variance (ANOVA) followed by Bonferroni test for multiple comparisons. ****p < 0.0001 for times ≥ 72 h. Histograms graphs show proliferation at 72 h across different treatments and concentrations. One‐way analysis of variance (ANOVA) followed by Bonferroni test for multiple comparisons. ns, not significant, **p < 0.0018, and ****p < 0.0001.
**Figure S2:** Representative images of migration of NRAS^Q61R^ EPCs at time = 0 h and time = 10 h after the insert was removed and the gap was created. Groups include no doxycycline and vehicle (No Dox + Vehicle), doxycycline and vehicle (Dox + Vehicle), and doxycycline (Dox) with RMC‐7977 (25 nM) added to the cell culture media. Representative images are shown, *n* = 3–6 wells per treatment. Scale bar = 275 μm.
**Figure S3:** Gap area of NRAS^Q61R^ EPCs was measured every 2 h from 0 to 10 h after creating the gap. Percentage gap closure was measured over time. Groups include no doxycycline and vehicle (No Dox + Vehicle), doxycycline and vehicle (Dox + Vehicle), and doxycycline (Dox) with RMC‐7977 (25 nM) added to the cell culture media. Data points are mean ± SD; *n* = 4 wells analysed. Statistical analysis of the time course was performed using two‐way Anova followed by Bonferroni test for multiple comparisons, **p < 0.0078 and ****p < 0.0001 over time. Histogram shows gap closure at 10 h for the study groups. For the histogram one‐way ANOVA was performed followed by Tukey's multiple comparison test. ns, not significant, **p < 0.0011, and ***p < 0.0006 at 10 h.
**Figure S4:** Representative images of NRAS^Q61R^ EPCs at 72 h. Groups include no doxycycline and vehicle (No Dox + Vehicle), doxycycline and vehicle, (Dox + Vehicle), and doxycycline with RMC‐7977 (Dox + 3.12, 6.25, 12.5, 25, 50, or 100 nM) added to the cell culture media. Representative images are shown. Scale bar = 400 μm.
**Figure S5:** Cell circularity index of RMC‐7977 dose response experiment in *NRAS* ^Q61R^ EPCs. Treatment groups included no doxycycline (−), doxycycline (+), and doxycycline (+) with RMC‐7977 (3.12, 6.25, 12.5, 25, 50, and 100 nM) added to the cell culture media for 72 h. Data points are mean ± SD. *n* = 3 wells per treatment. One‐way ANOVA was performed with Tukey's multiple comparison test. *p < 0.0157, **p < 0.0043, and ****p < 0.0001.
**Figure S6:** Cell circularity index of NRAS^WT^ and NRAS^Q61R^ EPCs treated with RMC‐7977. Treatment groups included no doxycycline (−), doxycycline (+), and doxycycline (+) with RMC‐7977 (3.12, 12.5, 50 nM) added to the cell culture media for 72 h. Data points are mean ± SD. *n* = 6 wells per treatment. One‐way ANOVA was performed with Tukey's multiple comparison test. ns, not significant, *p < 0.0224, and ****p < 0.0001.
**Figure S7:** Angiopoietin‐2 in cell culture medium of EPCs expressing *NRAS* ^Q61R^ measured using an ELISA. Treatment groups included no doxycycline and vehicle (−), doxycycline and vehicle (+), and doxycycline with different doses of RMC‐7977 (3.12–100 nM) added to the cell culture media for 72 h. Data points are mean ± SD. *n* = 3 wells per group. One‐way ANOVA was performed with Tukey's multiple comparison test. **p < 0.0027, ****p < 0.0001.
**Figure S8:** Representative images of vascular structures formed by *NRAS* ^Q61R^ EPCs treated with no doxycycline for 72 h and then after adding doxycycline to the cell culture media for 72 h. Representative images are shown, *n* = 4 wells analysed per treatment. Scale bar = 650 μm.
**Figure S9:** Representative images of vascular structures formed by *NRAS* ^Q61R^ EPCs between and around the beads on day 14. Images were taken using 4 by 4 tile scan (16 scans total). EPCs treatment groups included no doxycycline and vehicle (No Dox + Vehicle), doxycycline and vehicle (Dox + Vehicle), and doxycycline (Dox) in combination with RMC‐7977 (12.5, 25, or 50 nM). Representative images are shown, *n* = 4 wells analysed. 4× Scale bar = 650 μm.
**Figure S10:** Images of all xenografts plugs on day 11. Individual plugs were imaged after being harvested. Treatment groups included mice on no doxycycline diet treated with drug vehicle (No Dox + Vehicle), mice on doxycycline diet and treated with drug vehicle (Dox + Vehicle), and doxycycline (Dox) diet treated with RMC‐7977 (25 mg/kg). *n* = 4–13 mice per study group with 2 plugs per mouse. In two mice (W922 and W937) the plugs had joined and so were removed as one.
**Figure S11:** Immunohistochemical (IHC) staining was performed on xenograft plug sections using *Ulex europaeus* agglutinin‐I (UEA‐I), which labels human endothelial cells but not mouse endothelial cells. Treatment groups included: no doxycycline with vehicle control (No Dox + Vehicle), doxycycline with vehicle (Dox + Vehicle), and doxycycline (Dox) with RMC‐7977 (25 mg/kg). Images were acquired using the Lionheart Imaging System at 4× magnification (scale bar = 2000 μm) and 10× magnification (scale bar = 300 μm).
**Figure S12:** Mouse body weight (g) was measured during the study period. Treatment groups included mice on no doxycycline diet treated with drug vehicle (No Dox + Vehicle), mice on doxycycline diet and treated with drug vehicle (Dox + Vehicle), and doxycycline (Dox) diet treated with RMC‐7977 (25 mg/kg). Body weight in the no doxycycline treated with vehicle (No Dox + Vehicle) treated group decreased on days 1–3 due to dehydration. Hydrogel was provided, and body weight recovered thereafter. One‐way ANOVA was performed with Tukey's multiple comparison test. ns, not significant.
**Figure S13:** Schematic of the xenograft model protocol: Experimental timeline showing doxycycline (Dox) administration, the time of cell injection day (0), treatment period with RMC‐7977 or vehicle (day 5–12), and the time point for xenograft plug harvest (day 12).
**Figure S14:** Graphs of xenograft plug weights on day 12. Data points are individual plugs (left) and average plugs weight (right) with 2 plugs per mouse: 4 mice received no doxycycline treated with drug vehicle (No Dox + Vehicle), 13 mice on doxycycline‐containing diet treated with drug vehicle (Dox + Vehicle), and 12 mice on doxycycline diet and treated by oral gavage with RMC‐7977 (20 mg/kg; Dox + RMC‐7977). One‐way ANOVA followed by Tukey's multiple comparisons test for individual plugs. ns, not significant, *p < 0.0447, **p < 0.0014. Averaged plugs weight, ns, not significant, *p < 0.0311, **p < 0.0015.
**Figure S15:** Uncropped (raw) western blot images corresponding to Figure 1A showing phospho‐ERK (p‐ERK) and ERK (total) expression in EPCs expressing NRAS^Q61R^. Cells lysates were analysed at 72 h after treatment with no doxycycline (−), doxycycline (+), or doxycycline (+) in combination with RMC‐7977 (3.12, 6.25, 12.5, 25, 50, and 100 nM).
**Figure S16:** Uncropped (raw) western blot images corresponding to Figure 1A showing, phospho‐S6K (p‐S6K) and S6K (total) expression in EPCs expressing NRAS^Q61R^. Cells lysates were analysed at 72 h after treatment with no doxycycline (−), doxycycline (+), or doxycycline (+) in combination with RMC‐7977 (3.12, 6.25, 12.5, 25, 50, and 100 nM).
**Figure S17:** Uncropped (raw) western blot images corresponding to Figure 1A showing phospho‐AKT (p‐AKT) and AKT (total) expression in EPCs expressing NRAS^Q61R^. Cells lysates were analysed at 72 h after treatment with no doxycycline (−), doxycycline (+), or doxycycline (+) in combination with RMC‐7977 (3.12, 6.25, 12.5, 25, 50, and 100 nM).
**Figure S18:** Uncropped (raw) western blot images corresponding to Figure 1A showing Angiopoietin‐2 (ANG‐2) and actin expression in EPCs expressing NRAS^Q61R^. Cells lysates were analysed at 72 h after treatment with no doxycycline (−), doxycycline (+), or doxycycline (+) in combination with RMC‐7977 (3.12, 6.25, 12.5, 25, 50, and 100 nM).
**Figure S19:** Uncropped (raw) western blot images corresponding to Figure 1B showing NRAS^Q61R^, phospho‐ERK (p‐ERK), ERK (total), phospho‐S6K (p‐S6K), S6K (total), Angiopoietin‐2 (ANG‐2), and actin expression in EPCs expressing NRAS^WT^ and NRAS^Q61R^. Cells lysates were analysed at 72 h after treatment with no doxycycline (−), doxycycline (+), or doxycycline (+) in combination with RMC‐7977 (3.12, 12.5, 50 nM).
**Figure S20:** Uncropped (raw) western blot images corresponding to Figure 1B showing phospho‐AKT (p‐AKT) and AKT (total) expression in EPCs expressing NRAS^WT^ and NRAS^Q61R^. Cells lysates were analysed at 72 h after treatment with no doxycycline (−), doxycycline (+), or doxycycline (+) in combination with RMC‐7977 (3.12, 12.5, 50 nM).
**Figure S21:** Uncropped (raw) western blot images corresponding to Figure 1C (left) showing NRAS^Q61R^, phospho‐ERK (p‐ERK), ERK (total), phospho‐S6K (p‐S6K), S6K (total), phospho‐AKT (p‐AKT), AKT (total), and actin expression in EPCs expressing NRAS^Q61R^. Cells were treated with doxycycline for 48 h to induce NRAS^Q61R^ expression. Then media were changed with media containing no doxycycline (−), doxycycline (+), or doxycycline (+) in combination with RMC‐7977 (3.12 nM). Cell lysates were collected at 0.5, 1, 3, 6, 24, 48, 72 h. Antibodies specific to NRAS^Q61R^, phospho‐ERK (p‐ERK), ERK (total), phospho‐S6K (p‐S6K), S6K (total), phospho‐AKT (p‐AKT), AKT (total), Angiopoietin‐2 (ANG‐2), and actin were used.
**Figure S22:** Uncropped (raw) western blot images corresponding to Figure 1C (middle) showing NRAS^Q61R^, phospho‐ERK (p‐ERK), ERK (total), phospho‐S6K (p‐S6K), S6K (total), phospho‐AKT (p‐AKT), AKT (total), and actin expression in EPCs expressing NRAS^Q61R^. Cells were treated with doxycycline for 48 h to induce NRAS^Q61R^ expression. Then media were changed with media containing no doxycycline (−), doxycycline (+), or doxycycline (+) in combination with RMC‐7977 (12.5 nM). Cell lysates were collected at 0.5, 1, 3, 6, 24, 48, 72 h. Antibodies specific to NRAS^Q61R^, phospho‐ERK (p‐ERK), ERK (total), phospho‐S6K (p‐S6K), S6K (total), phospho‐AKT (p‐AKT), AKT (total), Angiopoietin‐2 (ANG‐2), and actin were used.
**Figure S23:** Uncropped (raw) western blot images corresponding to Figure 1C (right) showing NRAS^Q61R^, phospho‐ERK (p‐ERK), ERK (total), phospho‐S6K (p‐S6K), S6K (total), phospho‐AKT (p‐AKT), AKT (total), and actin expression in EPCs expressing NRAS^Q61R^. Cells were treated with doxycycline for 48 h to induce NRAS^Q61R^ expression. Then media were changed with media containing no doxycycline (−), doxycycline (+), or doxycycline (+) in combination with RMC‐7977 (50 nM). Cell lysates were collected at 0.5, 1, 3, 6, 24, 48, 72 h. Antibodies specific to NRAS^Q61R^, phospho‐ERK (p‐ERK), ERK (total), phospho‐S6K (p‐S6K), S6K (total), phospho‐AKT (p‐AKT), AKT (total), Angiopoietin‐2 (ANG‐2), and actin were used.
**Figure S24:** Densitometric analysis of western blot shown in Figure 1A RMC‐7977 Dose Response Experiment. Densitometric analysis of p‐ERK/ERK, ERK/actin, p‐S6K/S6K, S6K/actin, p‐AKT/AKT, AKT/actin, ANG‐2/actin protein expression in no doxycycline and doxycycline‐induced NRAS^Q61R^ endothelial progenitor cells (EPCs) treated with increasing concentrations of RMC‐7977 (3.12–100 nM) for 72 h. Band intensities were quantified using Multi Gauge Software and normalized to total ERK, total S6K, total AKT, or actin.
**Figure S25:** Densitometric analysis of western blot shown in Figure 1B NRAS^WT^ and NRAS^Q61R^ Treated with RMC (3.12, 12.5, 50 nM) Prevention Experiment. Densitometric analysis of NRAS^Q61R^/actin, p‐ERK/ERK, ERK/actin, p‐S6K/S6K, S6K/actin, p‐AKT/AKT, AKT/actin, ANG‐2/actin protein expression in no doxycycline and doxycycline‐induced NRAS^WT^ and NRAS^Q61R^ endothelial progenitor cells (EPCs) treated with increasing concentrations of RMC‐7977 (3.12, 12.5, and 50 nM) for 72 h. Band intensities were quantified using Multi Gauge Software and normalized to total ERK, total S6K, total AKT, or actin.
**Figure S26:** Densitometric analysis of western blot shown in Figure 1C (RMC 3.12 nM) time course. Densitometric analysis of NRAS^Q61R^/actin, p‐ERK/ERK, ERK/actin, p‐S6K/S6K, S6K/actin, p‐AKT/AKT, AKT/actin, ANG‐2/actin protein expression. Cells were treated with doxycycline (Dox) for 48 h to induce *NRAS* ^Q61R^ expression. The media was replaced with new media containing no doxycycline (−), doxycycline (+), and doxycycline (+) in combination with RMC‐7977 (3.12 nM) added to the cell culture media for 0.5, 1, 3, 6, 24, 48, and 72 h. Antibodies specific to p‐ERK, total ERK, p‐S6K, total S6K, p‐AKT, and total AKT, and actin were used. Band intensities were quantified using Multi Gauge Software and normalized to total ERK, total S6K, total AKT, or actin.
**Figure S27:** Densitometric analysis of western blot shown in Figure 1C (RMC 12.5 nM) time course. Densitometric analysis of NRAS^Q61R^/actin, p‐ERK/ERK, ERK/actin, p‐S6K/S6K, S6K/actin, p‐AKT/AKT, AKT/actin, ANG‐2/actin protein expression. Cells were treated with doxycycline (Dox) for 48 h to induce *NRAS* ^Q61R^ expression. The media was replaced with new media containing no doxycycline (−), doxycycline (+), and doxycycline (+) in combination with RMC‐7977 (12.5 nM) added to the cell culture media for 0.5, 1, 3, 6, 24, 48, and 72 h. Antibodies specific to p‐ERK, total ERK, p‐S6K, total S6K, p‐AKT, and total AKT, and actin were used. Band intensities were quantified using Multi Gauge Software and normalized to total ERK, total S6K, total AKT, or actin.
**Figure S28:** Densitometric analysis of western blot shown in Figure 1C (RMC 50 nM) time course. Densitometric analysis of NRAS^Q61R^/actin, p‐ERK/ERK, ERK/actin, p‐S6K/S6K, S6K/actin, p‐AKT/AKT, AKT/actin, ANG‐2/actin protein expression. Cells were treated with doxycycline (Dox) for 48 h to induce *NRAS* ^Q61R^ expression. The media was replaced with new media containing no doxycycline (−), doxycycline (+), and doxycycline (+) in combination with RMC‐7977 (50 nM) added to the cell culture media for 0.5, 1, 3, 6, 24, 48, and 72 h. Antibodies specific to p‐ERK, total ERK, p‐S6K, total S6K, p‐AKT, and total AKT, and actin were used. Band intensities were quantified using Multi Gauge Software and normalized to total ERK, total S6K, total AKT, or actin.

**Table S1:** Key Reagents for Western Blot and Staining.

**Table S2:** Key Reagents, kits, and plates for different experiments.

## Data Availability

The data support the findings of this study are available from the corresponding author upon reasonable request.
