# Supplementary material for: RAS Inhibitor RMC‐7977 Blocks Vascular Overgrowth of NRASQ61R Mutant Endothelial Cells
Source: J Cell Mol Med. 2026 Aug 10;30(15):e71316. doi: 10.1111/jcmm.71316 (PMC13458280; doi:10.1111/jcmm.71316)
Supplement: Supplementary file 1 [file JCMM-30-e71316-s003.pdf]

Supplementary Material for:

RAS Inhibitor RMC−7977 Blocks Vascular Overgrowth of *NRAS*^{Q61R} Mutant Endothelial Cells

Sara Alharbi[1,2,4], Svatava Merkle[2,4,5], Patricia Pastura[4], C. Griffin McDaniel[2,4], George S. Zaky[2,4], Andrew M. Waters[1,3], Timothy D. Le Cras[2,4]

Affiliations: [1]Department of Cancer Biology, University of Cincinnati College of Medicine, Cincinnati, Ohio; [2]Department of Pediatrics, University of Cincinnati College of Medicine; [3]Department of Surgery, University of Cincinnati College of Medicine; [4]Division of Pulmonary Biology, Cincinnati Children's Hospital Medical Center, Cincinnati, Ohio; [5]Division of Hematology, Cincinnati Children's Hospital Medical Center, Cincinnati, Ohio

Supplementary Data files:

Supplemental Figure S1.

Supplemental Figure S2.

Supplemental Figure S3.

Supplemental Figure S4.

Supplemental Figure S5.

Supplemental Figure S6.

Supplemental Figure S7.

Supplemental Figure S8.

Supplemental Figure S9.

Supplemental Figure S10.

Supplemental Figure S11.

Supplemental Figure S12.

Supplemental Figure S13.

Supplemental Figure S14.

Supplemental Figure S15.

Supplemental Figure S16.

Supplemental Figure S17.

Supplemental Figure S18.

Supplemental Figure S19.

Supplemental Figure S20.

Supplemental Figure S21.

Supplemental Figure S22.

Supplemental Figure S23.

Supplemental Figure S24.

Supplemental Figure S25.

Supplemental Figure S26.

Supplemental Figure S27.

Supplemental Figure S28.

Supplemental Table S1.

Supplemental Table S2.

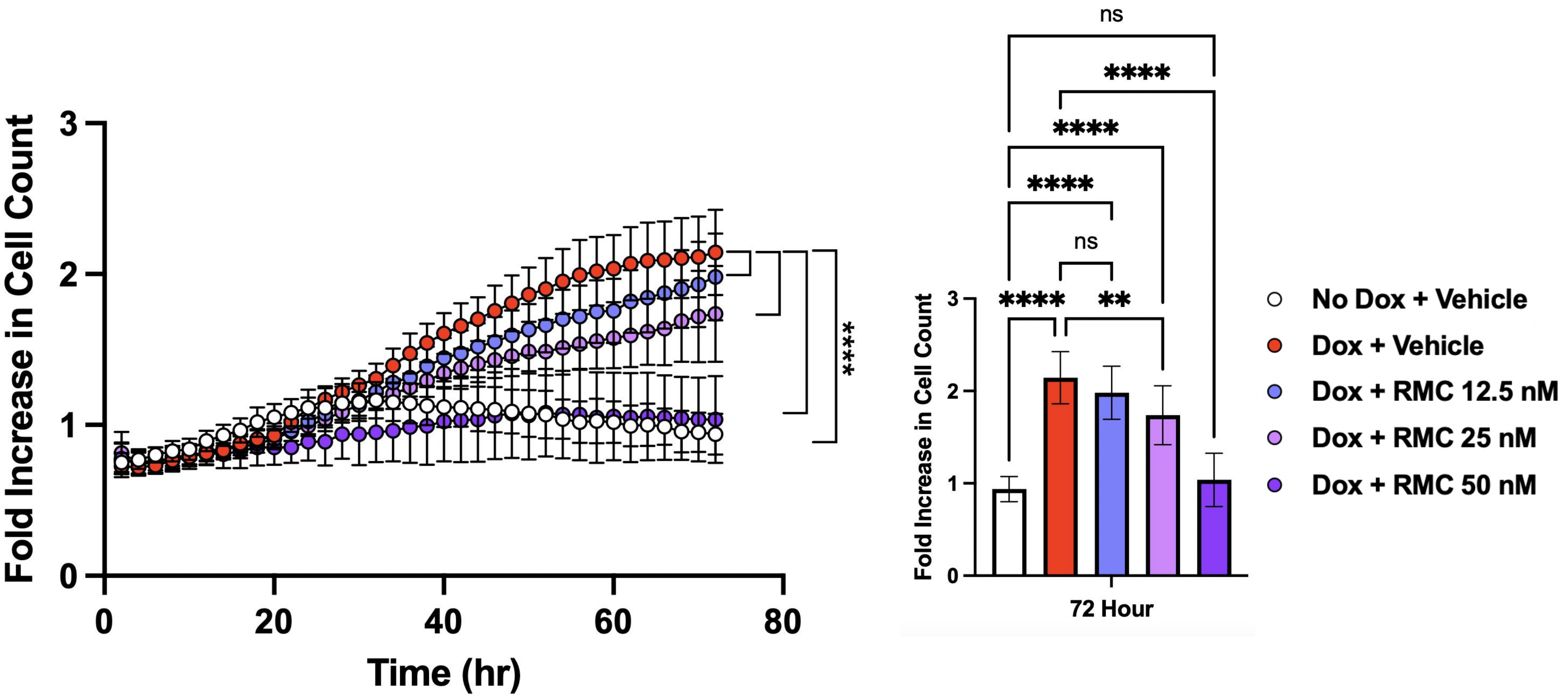

Supplemental Figure S1: RMC−7977 dose–response analysis of proliferation in EPCs expressing NRAS^{Q61R}. Cell counts were measured using a Lionheart automated microscope system every 2 h for 72 h. EPCs groups included no doxycycline and vehicle (No Dox + Vehicle), doxycycline and vehicle (Dox + Vehicle), and doxycycline with RMC−7977 (Dox + RMC 12.5, 25, or 50 nM) added to the cell culture media. Data points are mean ± SD; n=16 wells per treatment. Two−way analysis of variance (ANOVA) followed by Bonferroni test for multiple comparisons. **** $p<0.0001$ for times ≥ 72 h. Histograms graphs show proliferation at 72 h across different treatments and concentrations. One−way analysis of variance (ANOVA) followed by Bonferroni test for multiple comparisons. ns= not significant, ** $p<0.0018$, and **** $p<0.0001$.

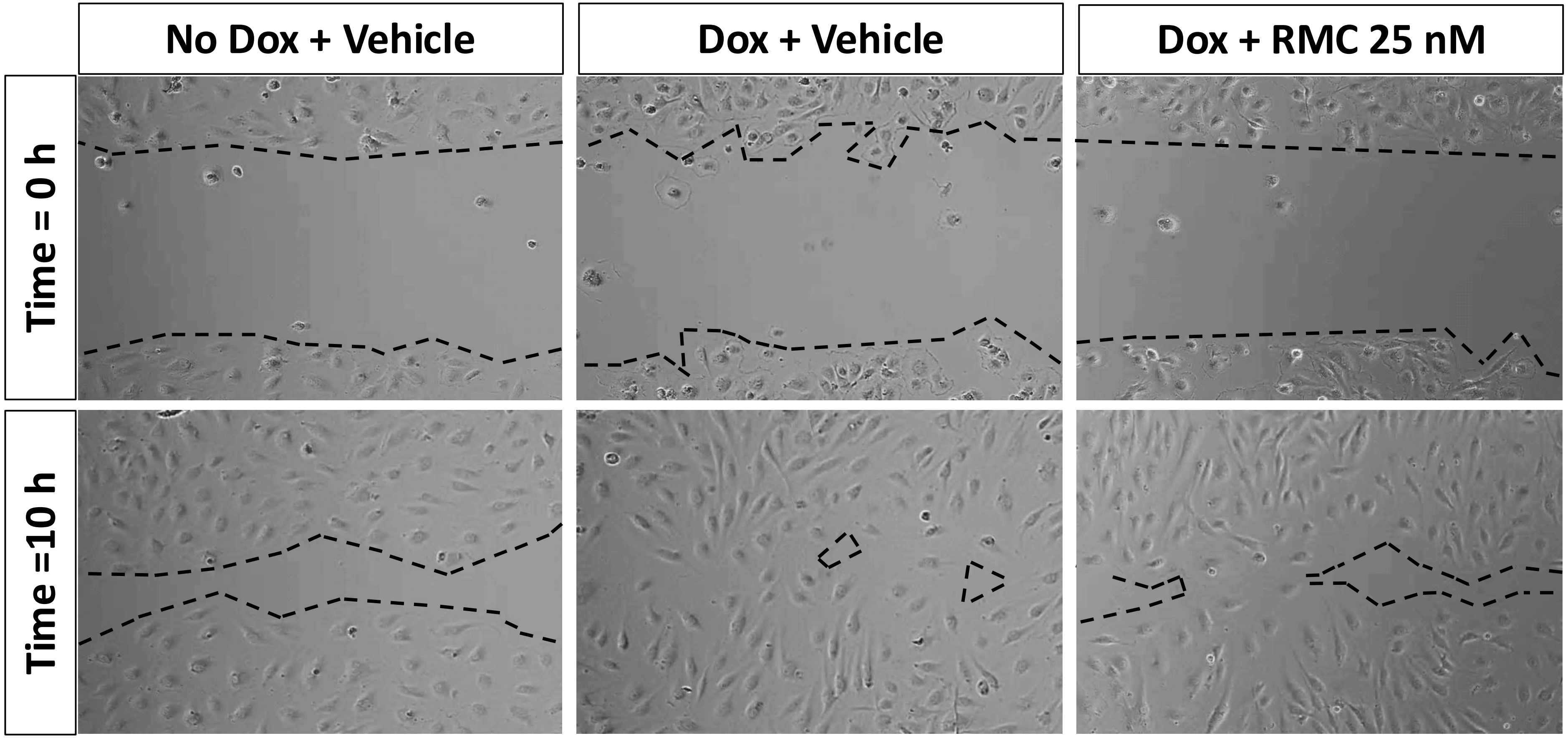

Supplemental Figure S2: Representative images of migration of NRAS^{Q61R} EPCs at time =0 h and time=10 h after the insert was removed and the gap was created. Groups include no doxycycline and vehicle (No Dox + Vehicle), doxycycline and vehicle (Dox + Vehicle), and doxycycline (Dox) with RMC−7977 (25 nM) added to the cell culture media. Representative images are shown, n=3−6 wells per treatment. Scale bar =275 µm.

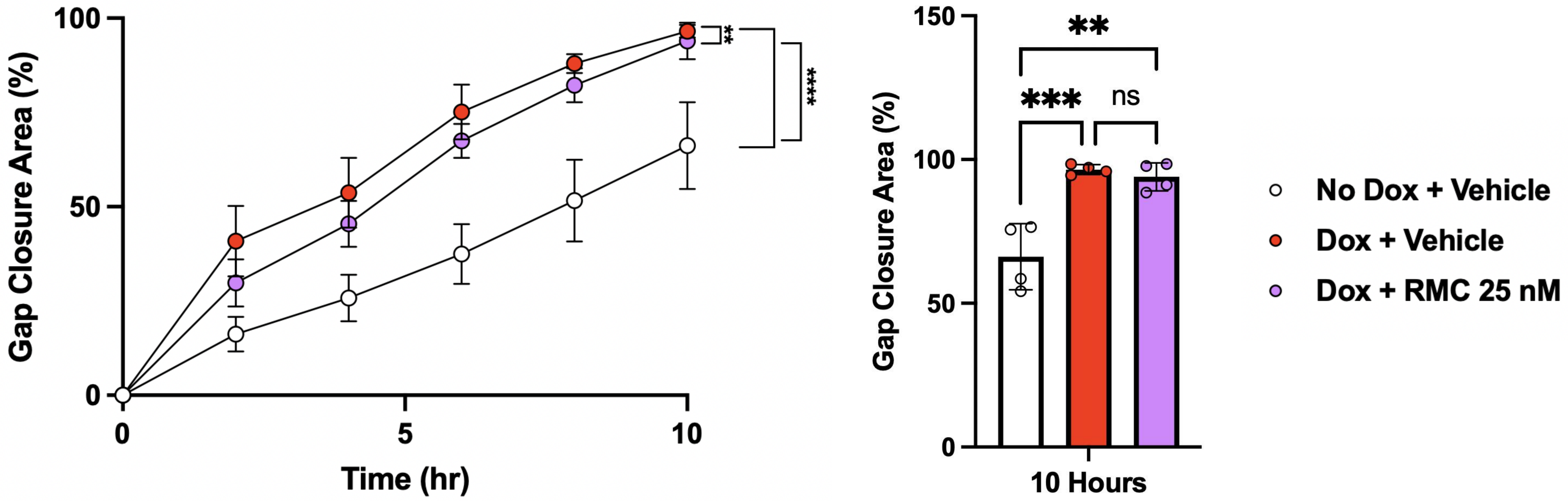

Supplemental Figure S3: Gap area of NRAS^{Q61R} EPCs was measured every 2 h from 0 to 10 hours after creating the gap. Percentage gap closure was measured over time. Groups include no doxycycline and vehicle (No Dox + Vehicle), doxycycline and vehicle (Dox + Vehicle), and doxycycline (Dox) with RMC−7977 (25 nM) added to the cell culture media. Data points are mean ± SD; n= 4 wells analyzed. Statistical analysis of the time course was performed using two−way Anova followed by Bonferroni test for multiple comparisons, ** $p < 0.0078$ and ****$p < 0.0001$ over time. Histogram shows gap closure at 10 hours for the study groups. For the histogram one–way ANOVA was performed followed by Tukey's multiple comparison test. ns= not significant, ** $p<0.0011$, and ***$p<0.0006$ at 10 hours.

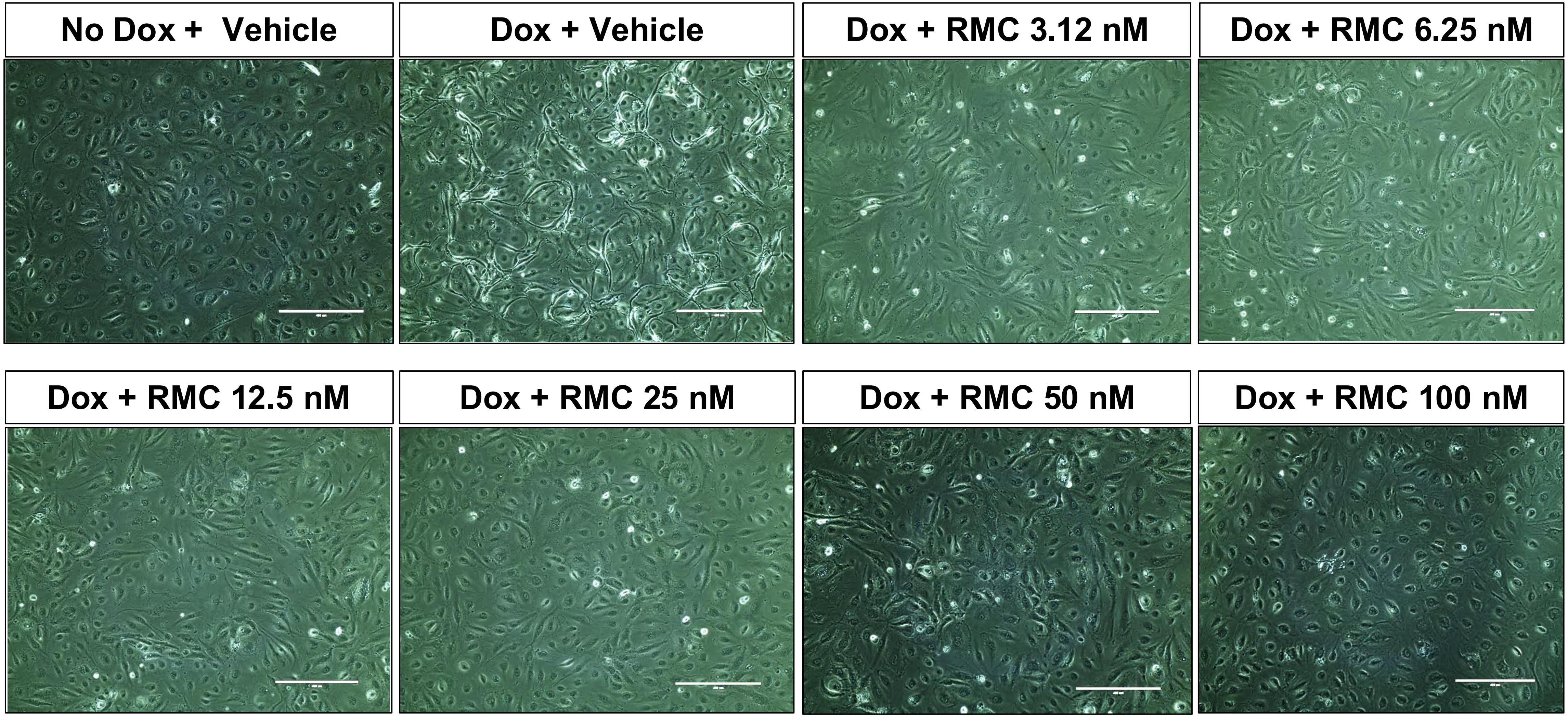

Supplemental Figure S4: Representative images of NRAS^{Q61R} EPCs at 72 h. Groups include no doxycycline and vehicle (No Dox + Vehicle), doxycycline and vehicle, (Dox + Vehicle), and doxycycline with RMC−7977 (Dox + 3.12, 6.25, 12.5, 25, 50, or 100 nM) added to the cell culture media. Representative images are shown. Scale bar =400 μm.

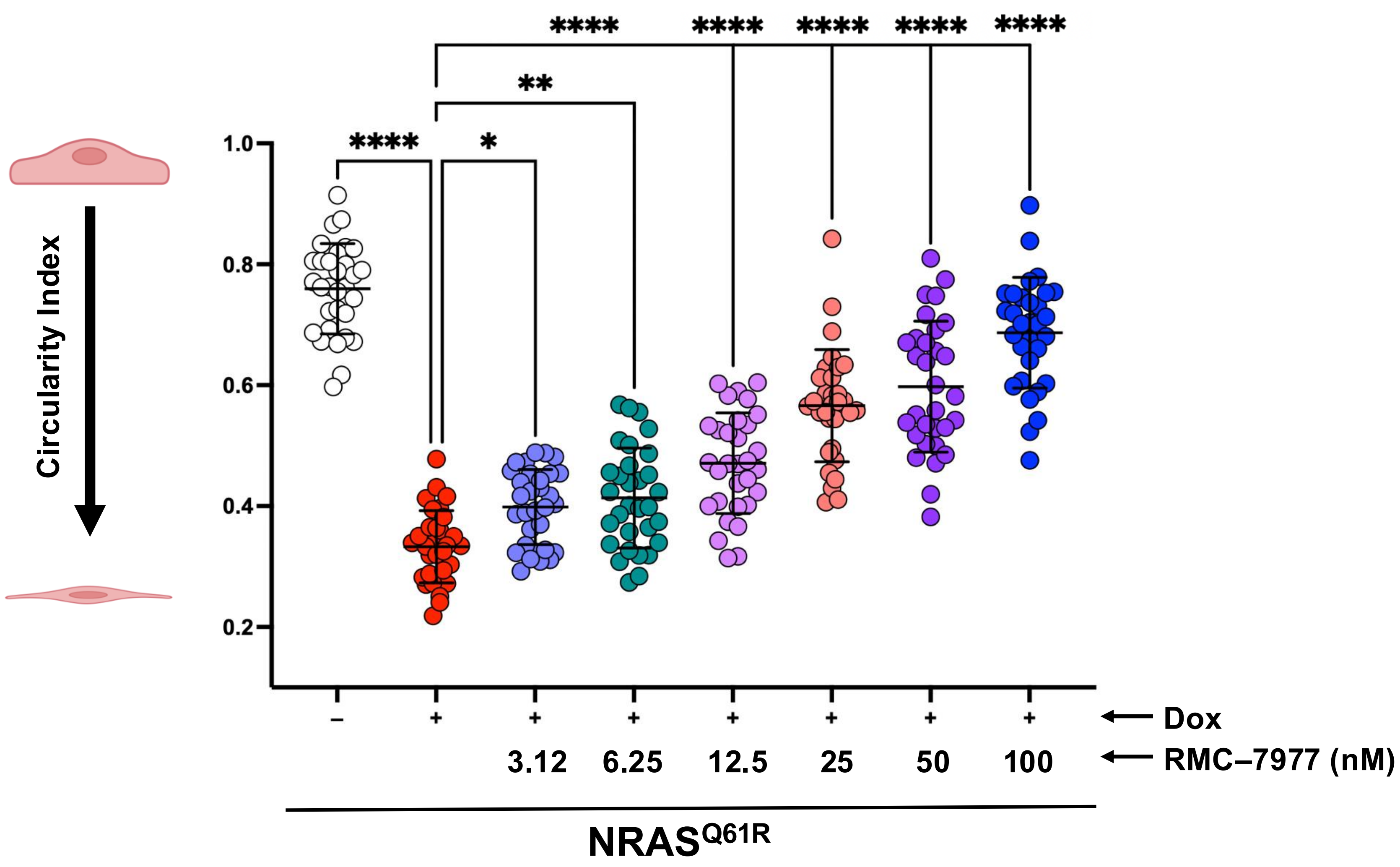

Supplemental Figure S5: Cell circularity index of RMC−7977 dose response experiment in *NRAS*^{Q61R} EPCs. Treatment groups included no doxycycline (−), doxycycline (+), and doxycycline (+) with RMC−7977 (3.12, 6.25, 12.5, 25, 50, and 100 nM) added to the cell culture media for 72 h. Data points are mean ± SD. n=3 wells per treatment. One−way ANOVA was performed with Tukey's multiple comparison test. *p <0.0157, **p <.0043, and ****p < .0001.

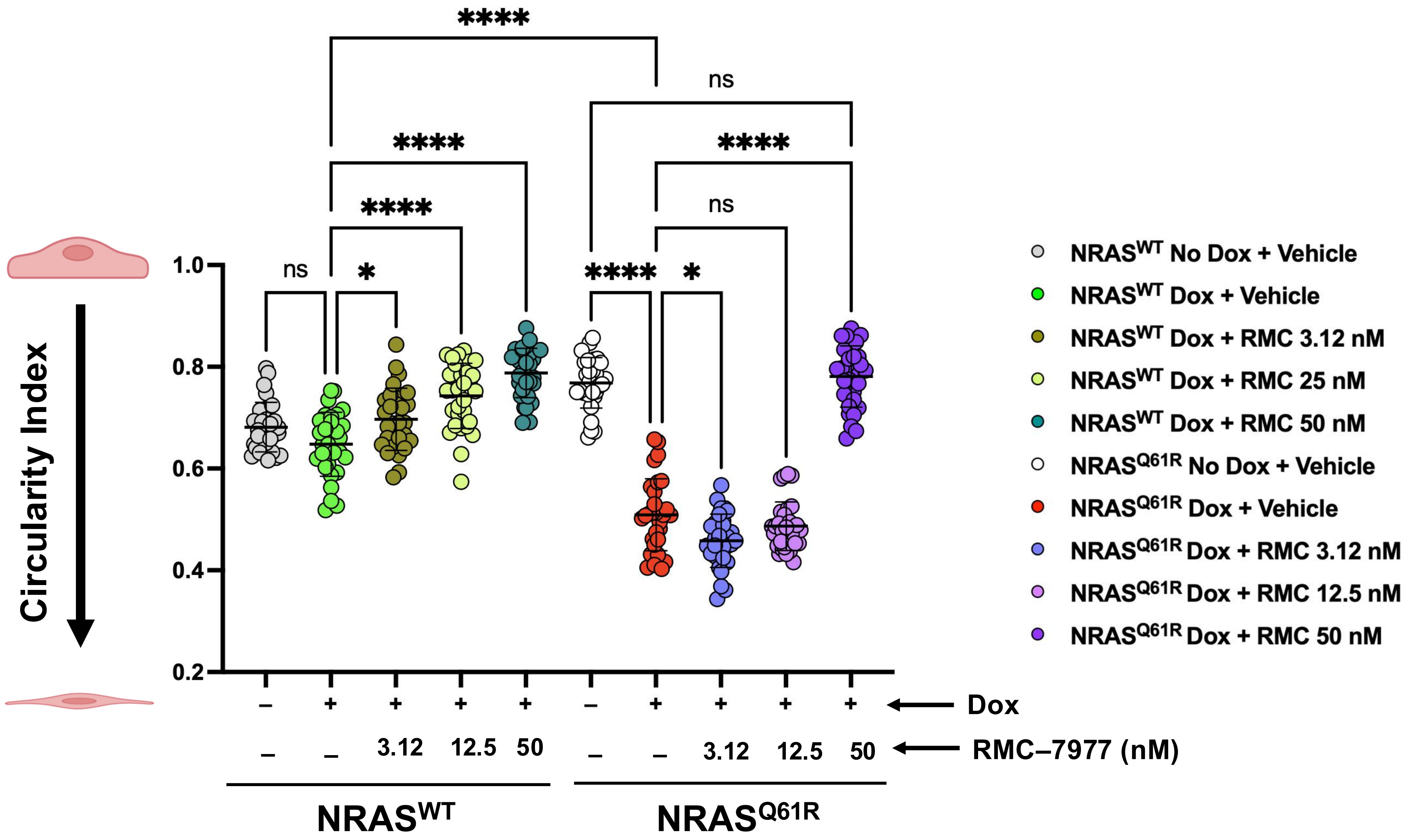

Supplemental Figure S6: Cell circularity index of $NRAS^{WT}$ and $NRAS^{Q61R}$ EPCs treated with RMC−7977. Treatment groups included no doxycycline (−), doxycycline (+), and doxycycline (+) with RMC−7977 (3.12, 12.5, 50 nM) added to the cell culture media for 72 h. Data points are mean ± SD. n=6 wells per treatment. One−way ANOVA was performed with Tukey's multiple comparison test. ns= not significant, *p < .0224, and ****p < .0001.

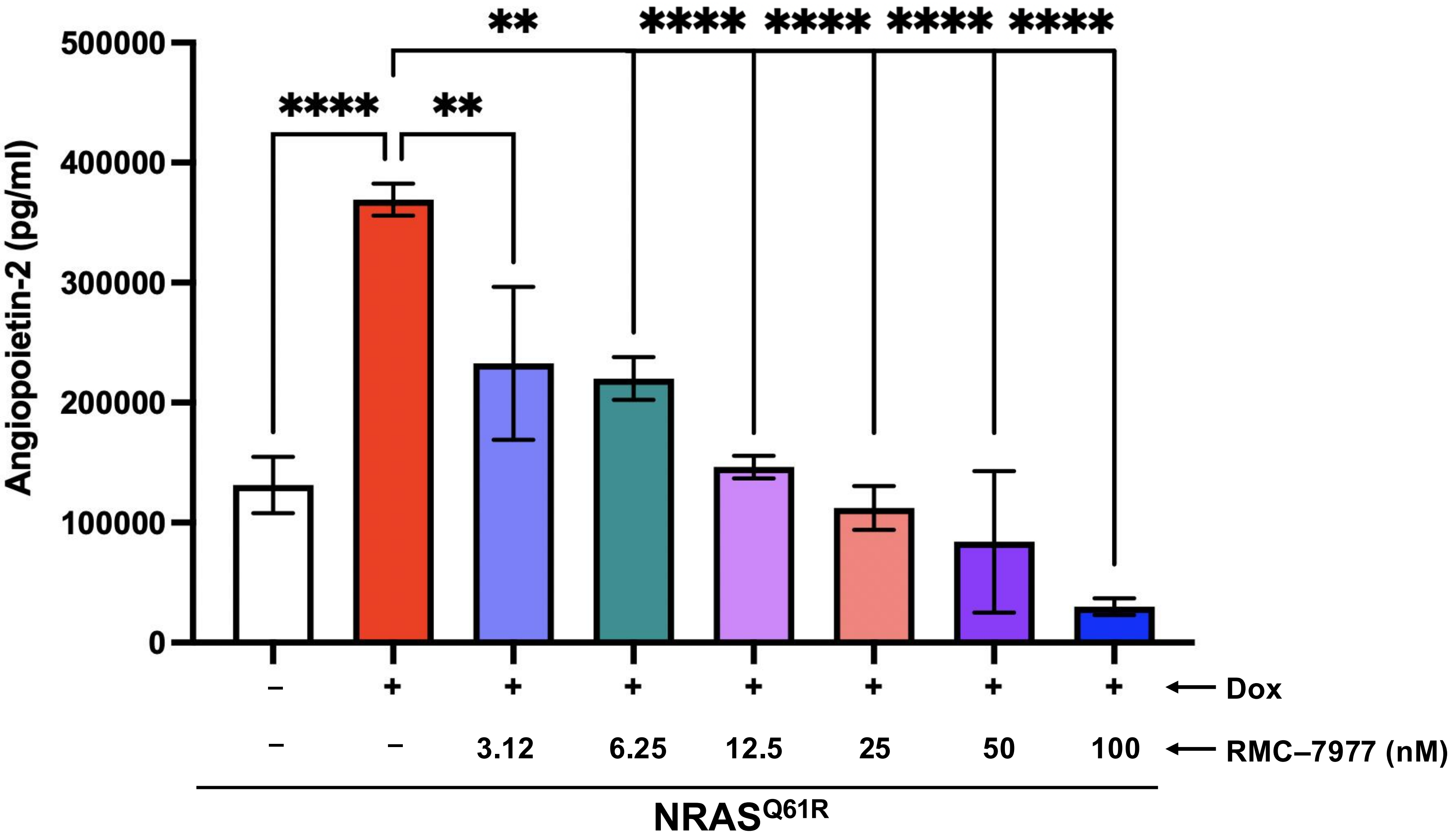

Supplemental Figure S7: Angiopoietin−2 in cell culture medium of EPCs expressing *NRAS*^{Q61R} measured using an ELISA. Treatment groups included no doxycycline and vehicle (−), doxycycline and vehicle (+), and doxycycline with different doses of RMC−7977 (3.12-100 nM) added to the cell culture media for 72 h. Data points are mean ± SD. n=3 wells per group. One−way ANOVA was performed with Tukey's multiple comparison test. ** $p < 0.0027$, ****$p < 0.0001$.

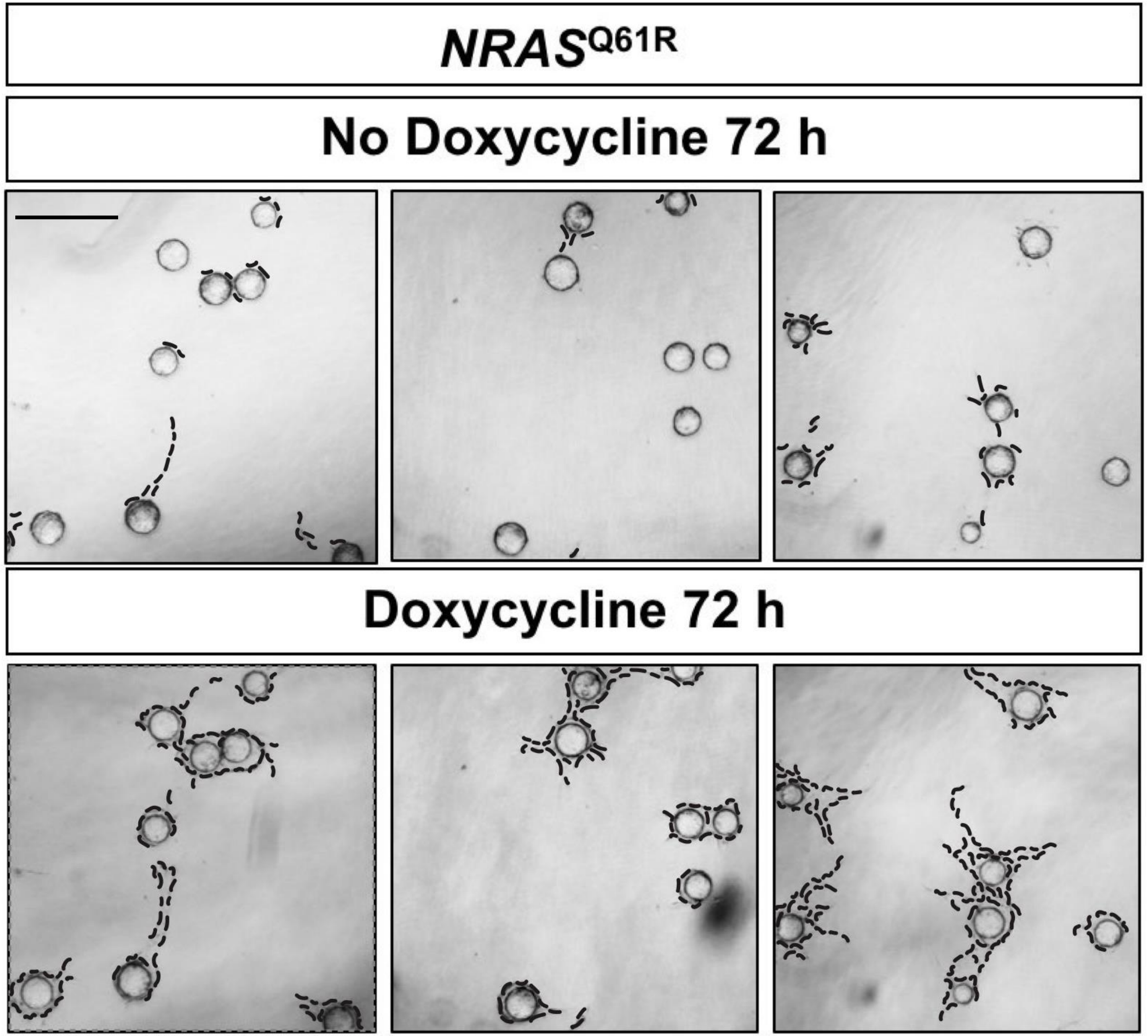

Supplemental Figure S8: Representative images of vascular structures formed by *NRAS*^{Q61R} EPCs treated with no doxycycline for 72 hours and then after adding doxycycline to the cell culture media for 72 hours. Representative images are shown, n=4 wells analyzed per treatment. Scale bar=650 μm.

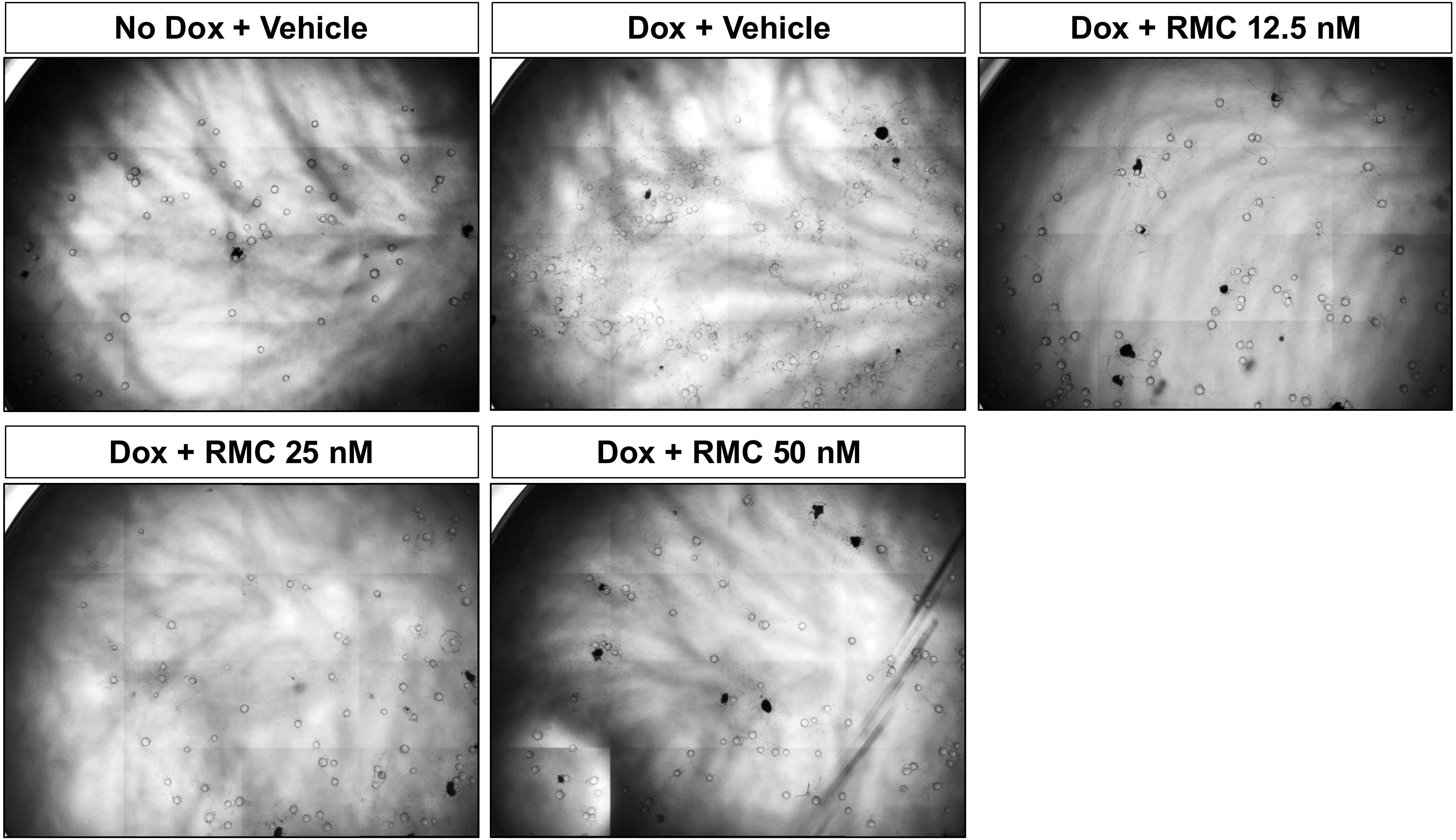

Supplemental Figure S9: Representative images of vascular structures formed by *NRAS*^{Q61R} EPCs between and around the beads on day 14. Images were taken using 4 by 4 tile scan (16 scans total). EPCs treatment groups included no doxycycline and vehicle (No Dox + Vehicle), doxycycline and vehicle (Dox + Vehicle), and doxycycline (Dox) in combination with RMC−7977 (12.5, 25, or 50 nM). Representative images are shown, n=4 wells analyzed. 4x Scale bar=650 µm.

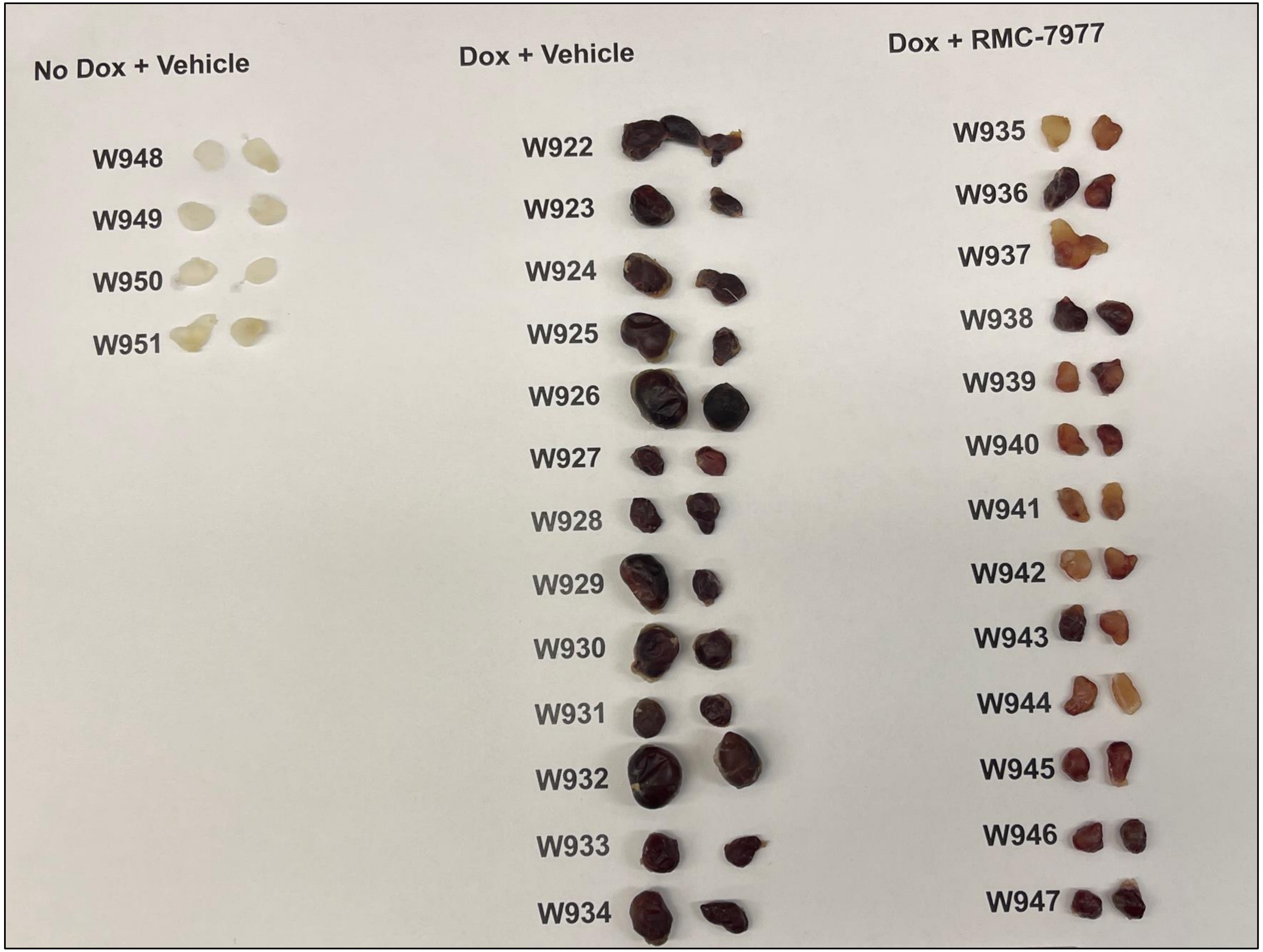

Supplemental Figure S10: Images of all xenograft plugs on day 11. Individual plugs were imaged after being harvested. Treatment groups included mice on no doxycycline diet treated with drug vehicle (No Dox + Vehicle), mice on doxycycline diet and treated with drug vehicle (Dox + Vehicle), and doxycycline (Dox) diet treated with RMC−7977 (25 mg/kg). n= 4−13 mice per study group with 2 plugs per mouse. In two mice (W922 and W937) the plugs had joined and so were removed as one.

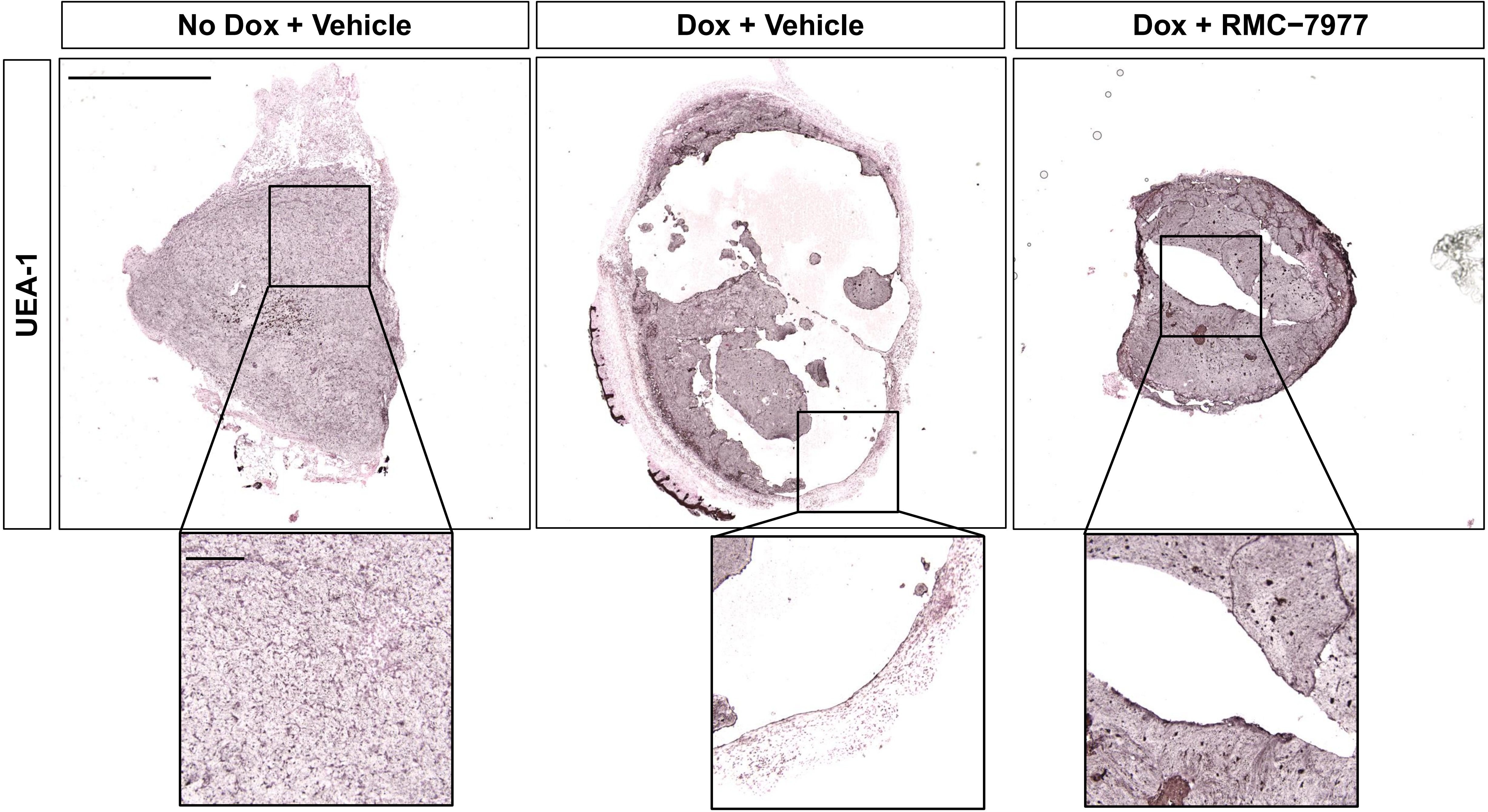

Supplemental Figure S11: Immunohistochemical (IHC) staining was performed on xenograft plug sections using *Ulex europaeus* agglutinin-I (UEA-I), which labels human endothelial cells but not mouse endothelial cells. Treatment groups included: no doxycycline with vehicle control (No Dox + Vehicle), doxycycline with vehicle (Dox + Vehicle), and doxycycline with RMC–7977 (25 mg/kg). Images were acquired using the Lionheart Imaging System at 4x magnification (scale bar = 2000 µm) and 10x magnification (scale bar = 300 µm).

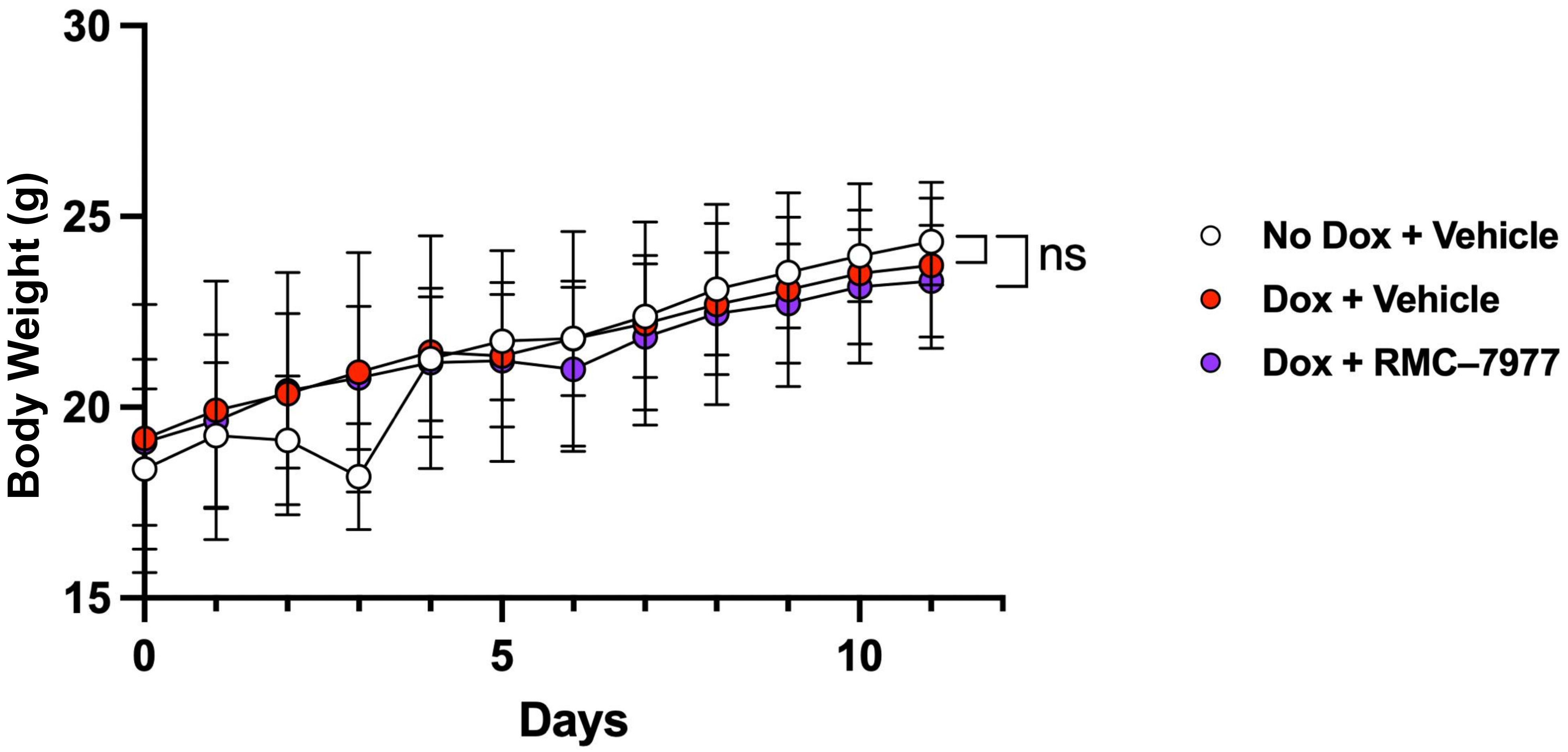

Supplemental Figure S12: Mouse body weight (g) was measured during the study period. Treatment groups included mice on no doxycycline diet treated with drug vehicle (No Dox + Vehicle), mice on doxycycline diet and treated with drug vehicle (Dox + Vehicle), and doxycycline (Dox) diet treated with RMC−7977 (25 mg/kg). Body weight in the No Dox Vehicle treated group decreased on days 1−3 due to dehydration. Hydrogel was provided, and body weight recovered thereafter. One−way ANOVA was performed with Tukey's multiple comparison test. ns= not significant.

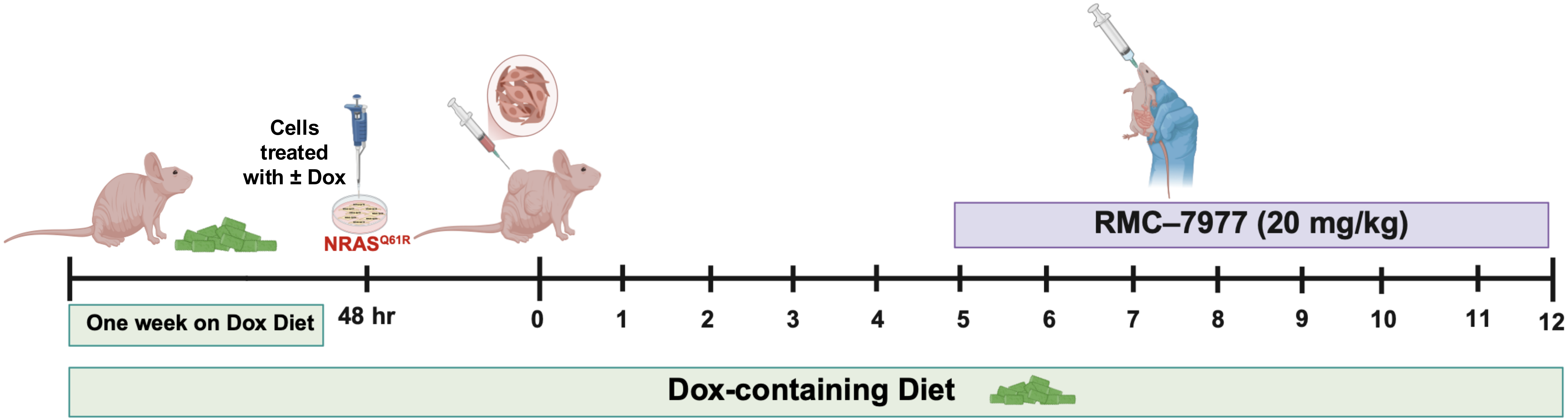

Supplemental Figure S13: Schematic of the xenograft model protocol: Experimental timeline showing doxycycline (Dox) administration, the time of cell injection day (0), treatment period with RMC−7977 or vehicle (day 5−12), and the time point for xenograft plug harvest (day 12).

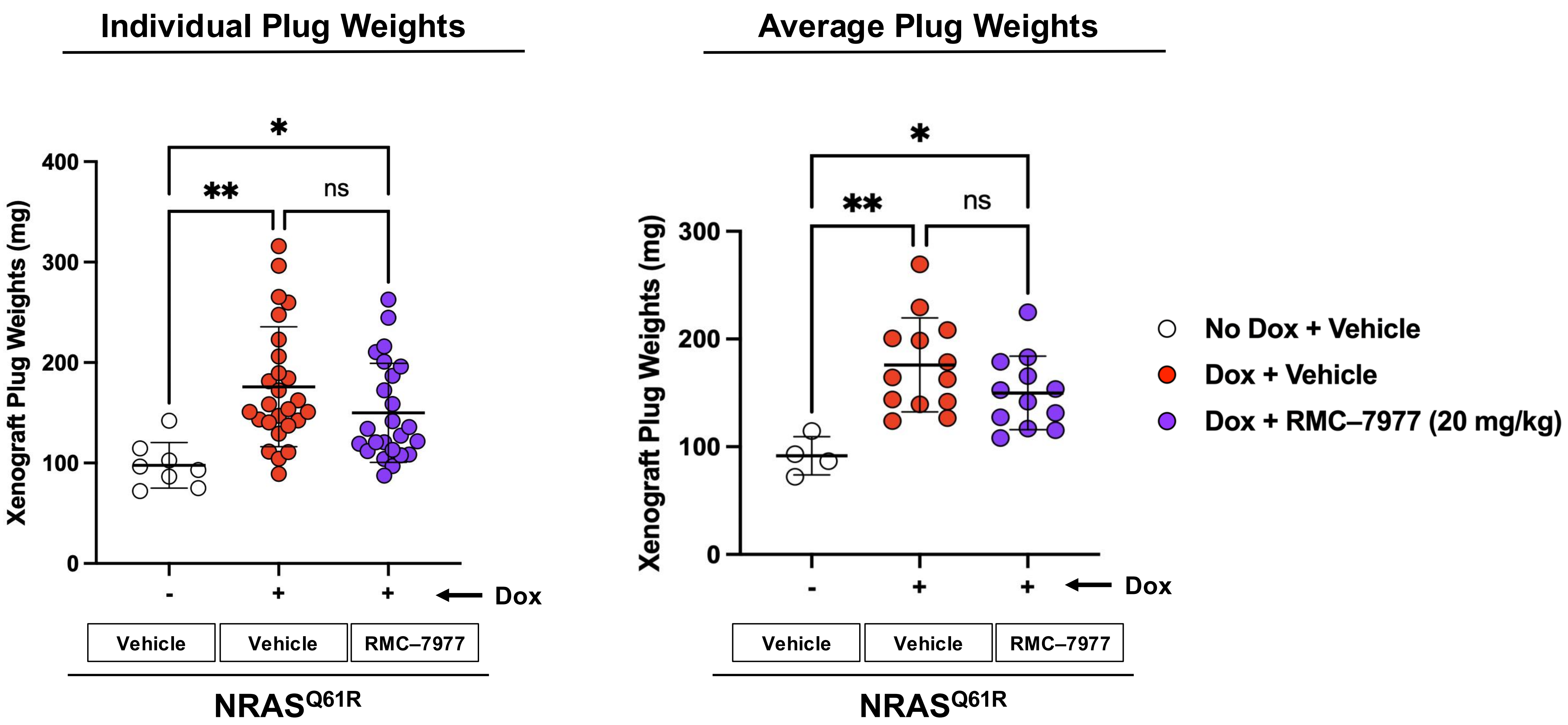

Supplemental Figure S14: Graphs of xenograft plug weights on day 12. Data points are individual plugs (left) and average plugs weight (right) with 2 plugs per mouse: 4 mice received no doxycycline (No Dox + Vehicle), 13 mice on doxycycline-containing diet (Dox + Vehicle), and 12 mice doxycycline diet and treated by gavage with RMC–7977 (20 mg/kg; Dox + RMC−7977). One way ANOVA followed by Tukey's multiple comparisons test for individual plugs. ns= not significant, *p < .0447, **P < .0014. Averaged plugs weight, ns= not significant, *p < .0311, **P < .0015.

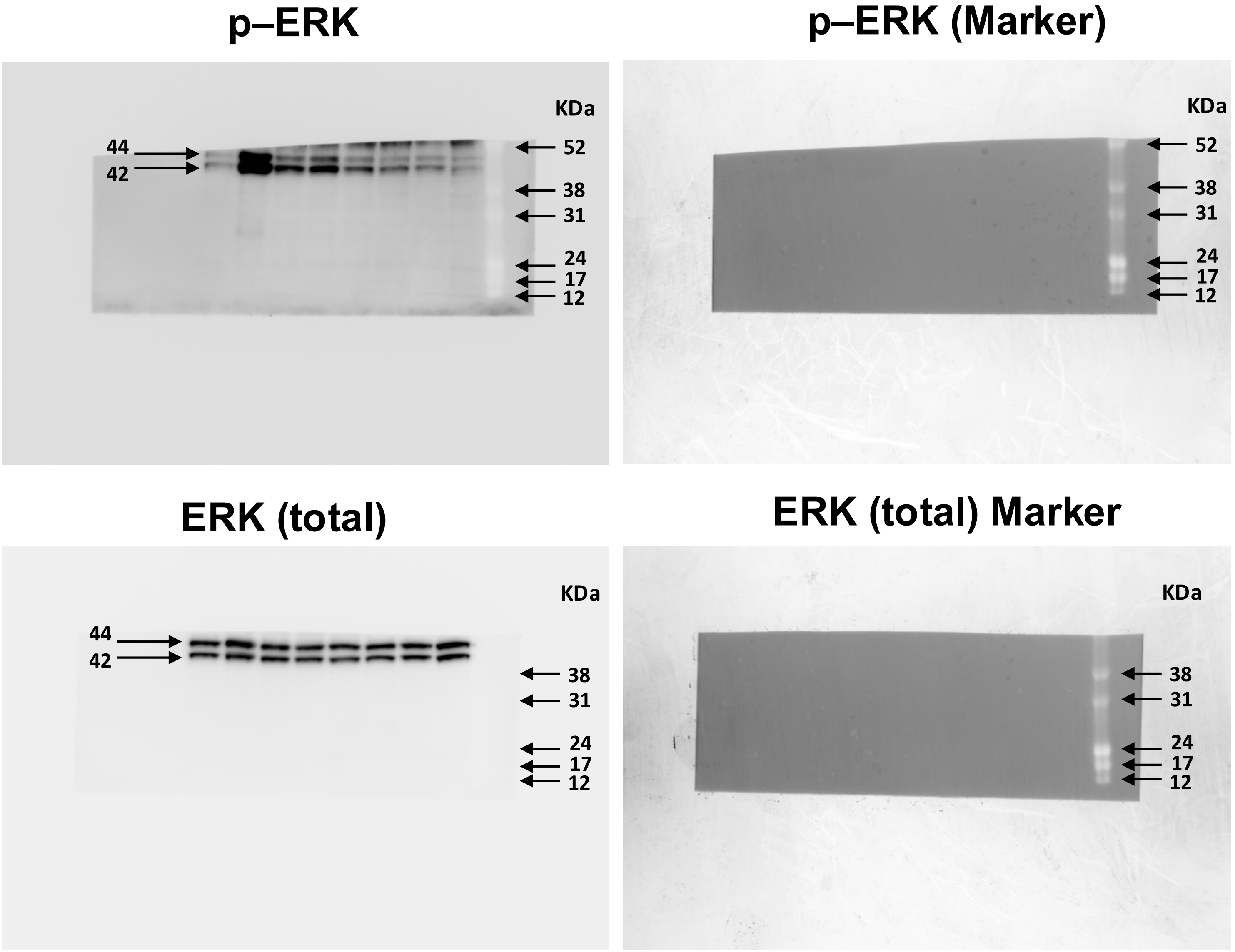

Supplemental Figure S15: Uncropped (raw) western blot images corresponding to Figure 1A showing p−ERK and total ERK expression in EPCs expressing NRAS^{Q61R}. Cells lysates were analyzed at 72 h after treatment with no doxycycline (−), doxycycline (+), or doxycycline (+) in combination with RMC−7977 (3.12, 6.25, 12.5, 25, 50, and 100 nM).

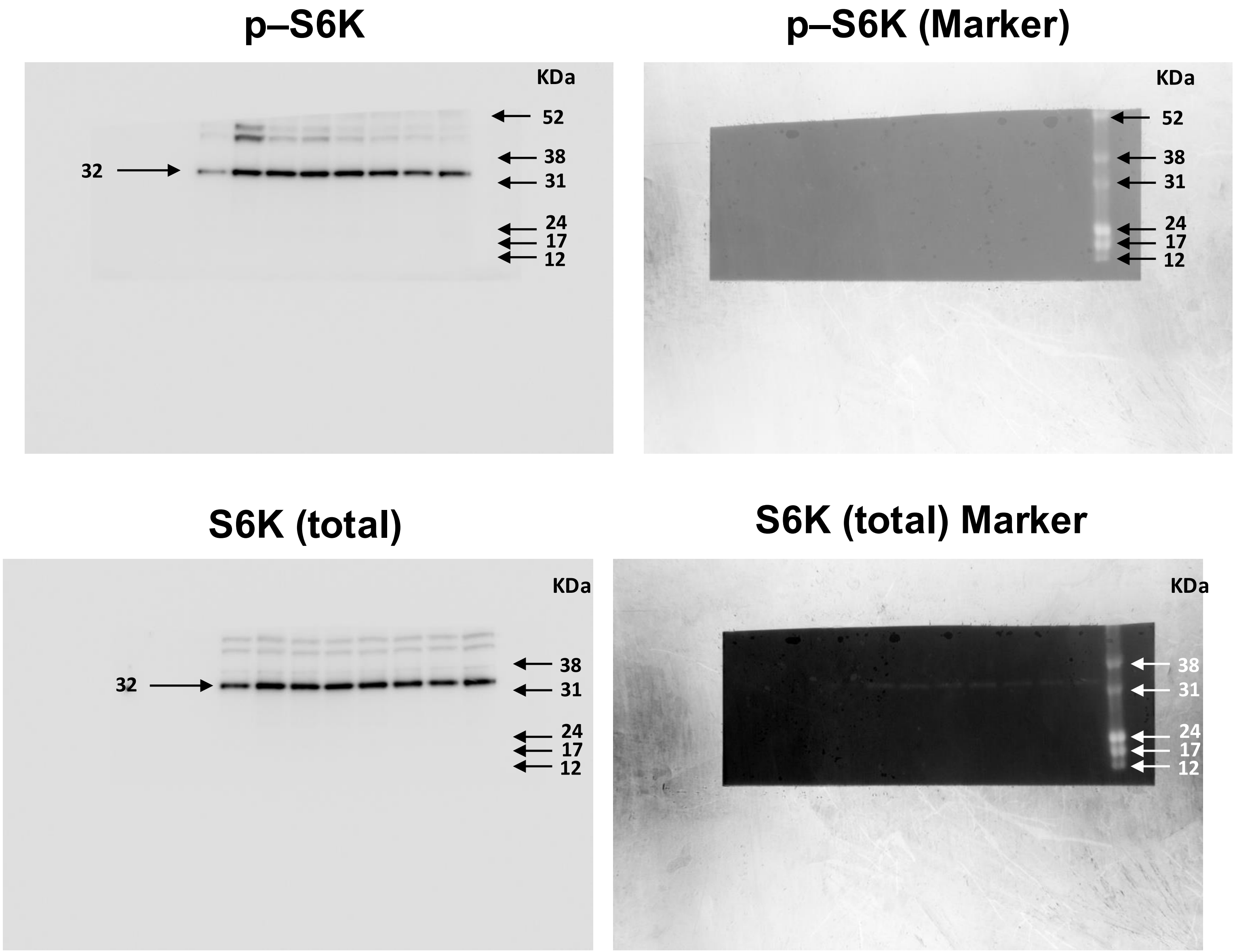

Supplemental Figure S16: Uncropped (raw) western blot images corresponding to Figure 1A showing, p−S6K and total S6K expression in EPCs expressing $NRAS^{Q61R}$. Cells lysates were analyzed at 72 h after treatment with no doxycycline (−), doxycycline (+), or doxycycline (+) in combination with RMC−7977 (3.12, 6.25, 12.5, 25, 50, and 100 nM).

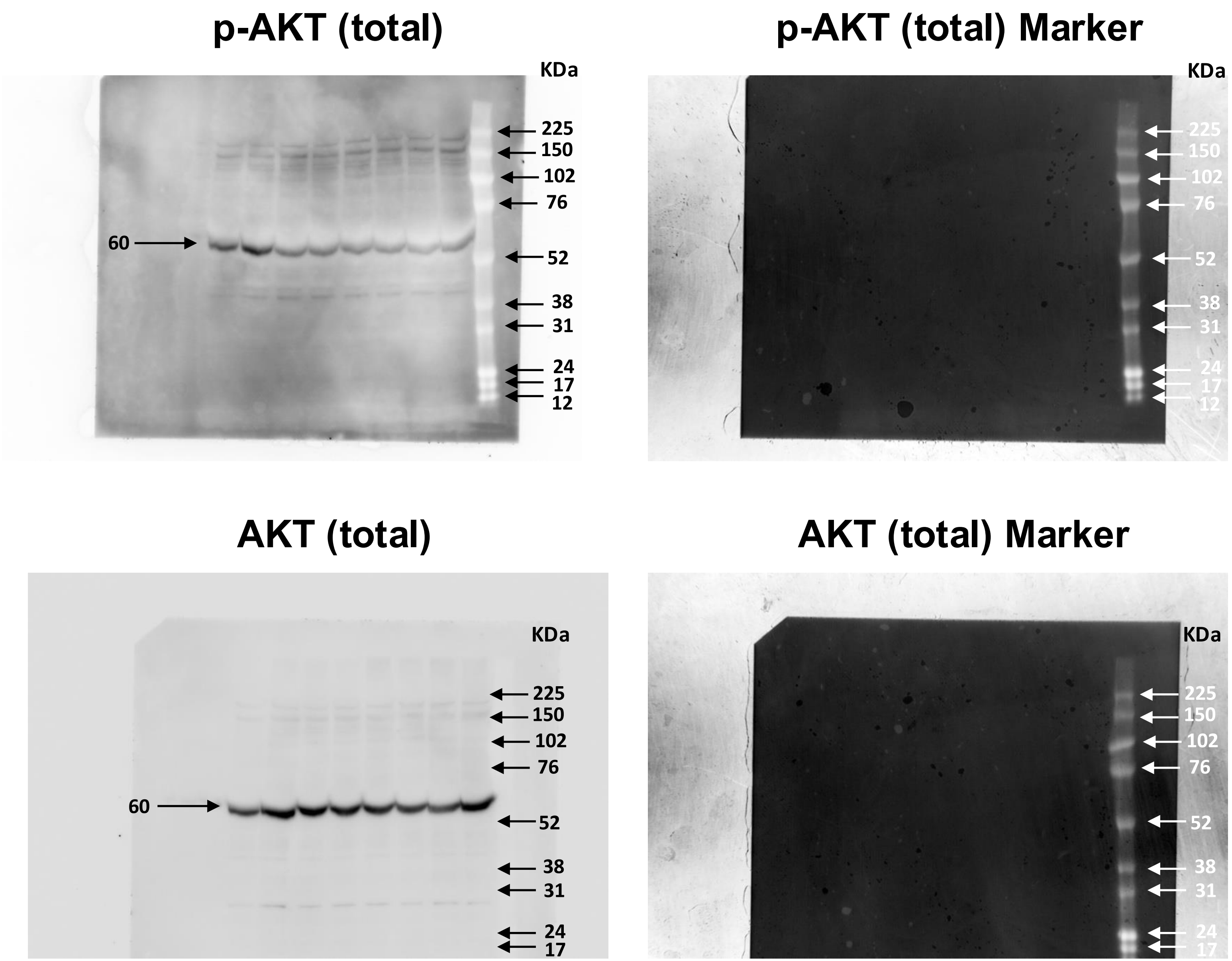

Supplemental Figure S17: Uncropped (raw) western blot images corresponding to Figure 1A showing p-AKT and AKT (total) expression in EPCs expressing $NRAS^{Q61R}$. Cells lysates were analyzed at 72 h after treatment with no doxycycline (−), doxycycline (+), or doxycycline (+) in combination with RMC−7977 (3.12, 6.25, 12.5, 25, 50, and 100 nM).

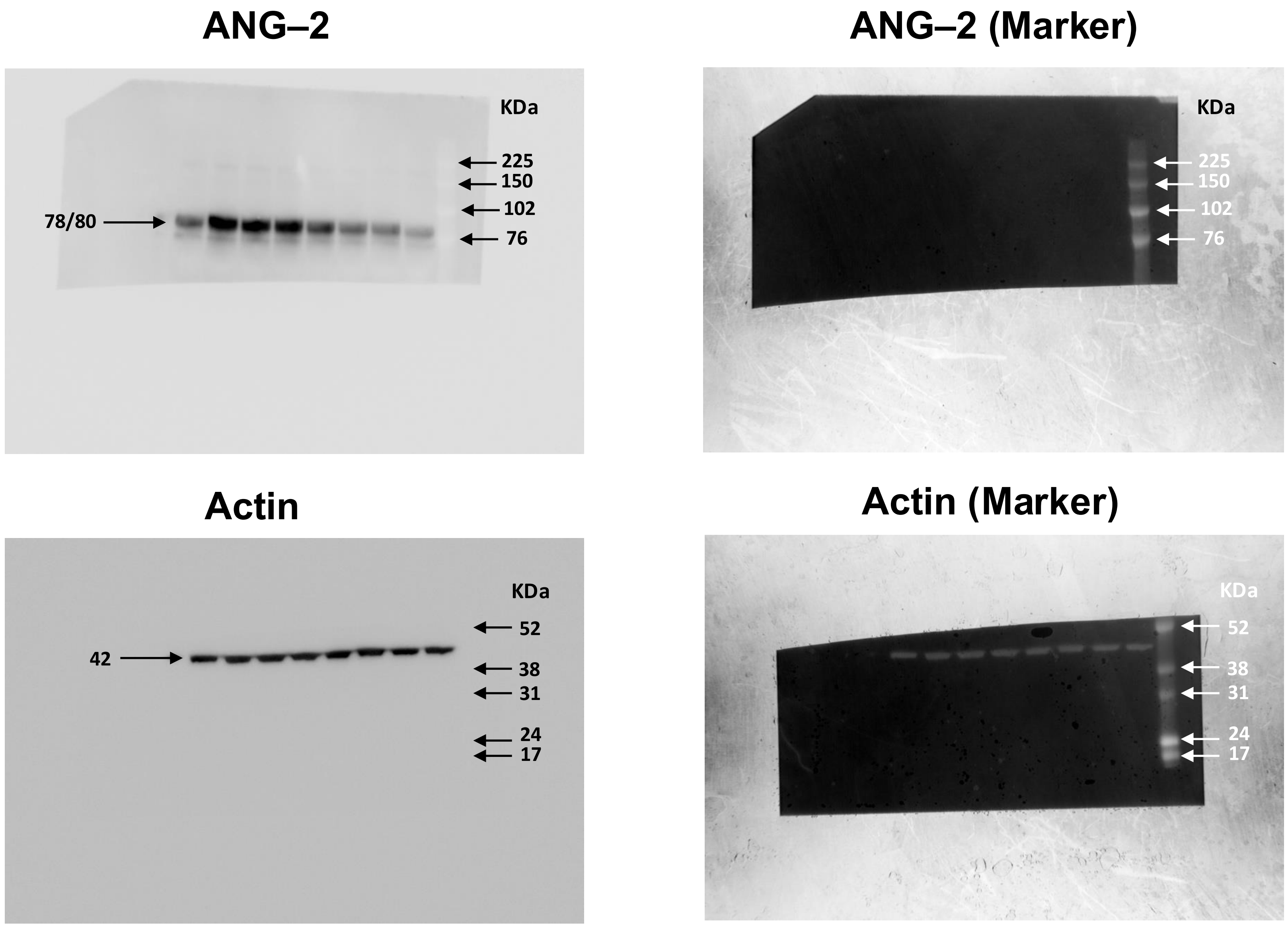

Supplemental Figure S18: Uncropped (raw) western blot images corresponding to Figure 1A showing ANG−2 and actin expression in EPCs expressing NRAS^{Q61R}. Cells lysates were analyzed at 72 h after treatment with no doxycycline (−), doxycycline (+), or doxycycline (+) in combination with RMC−7977 (3.12, 6.25, 12.5, 25, 50, and 100 nM).

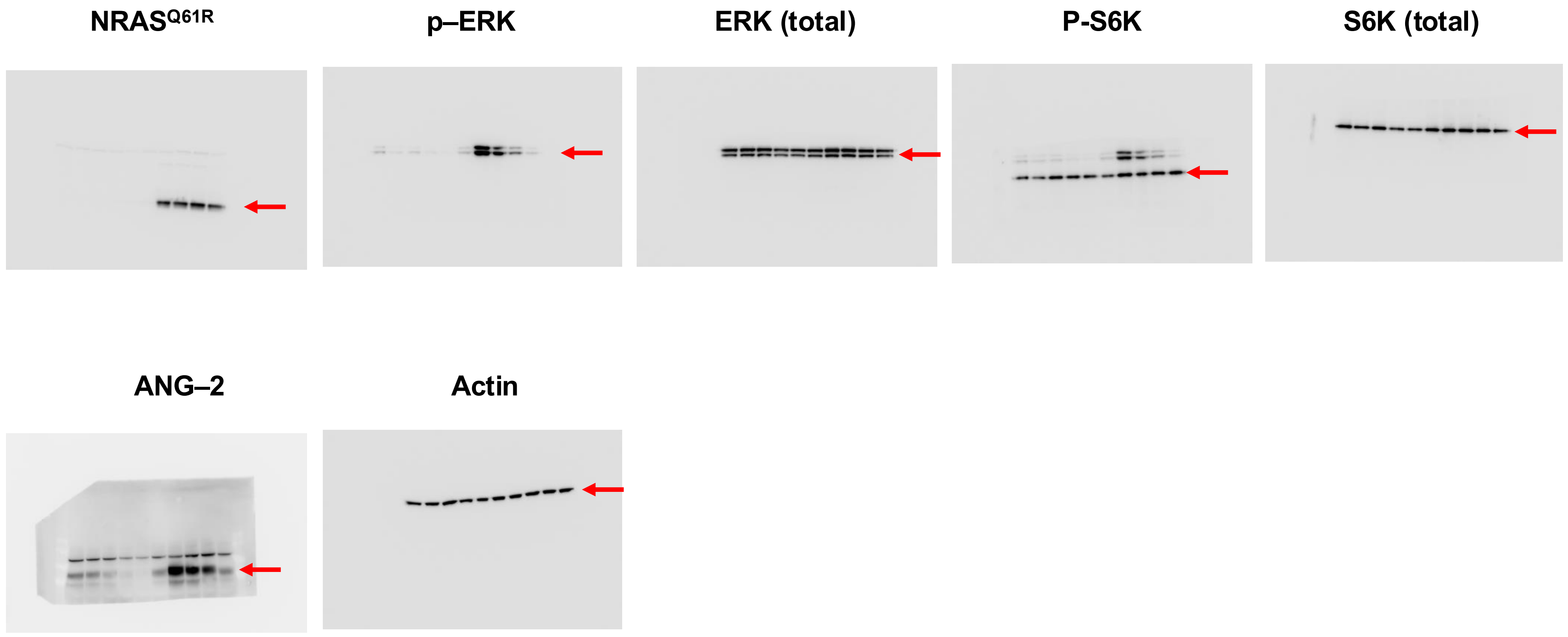

Supplemental Figure S19: Uncropped (raw) western blot images corresponding to Figure 1B showing NRAS^{Q61R}, p–ERK, total ERK, p–S6K, total S6K, ANG–2, and actin expression in EPCs expressing NRAS^{WT} and NRAS^{Q61R}. Cells lysates were analyzed at 72 h after treatment with no doxycycline (−), doxycycline (+), or doxycycline (+) in combination with RMC−7977 (3.12, 12.5, 50 nM).

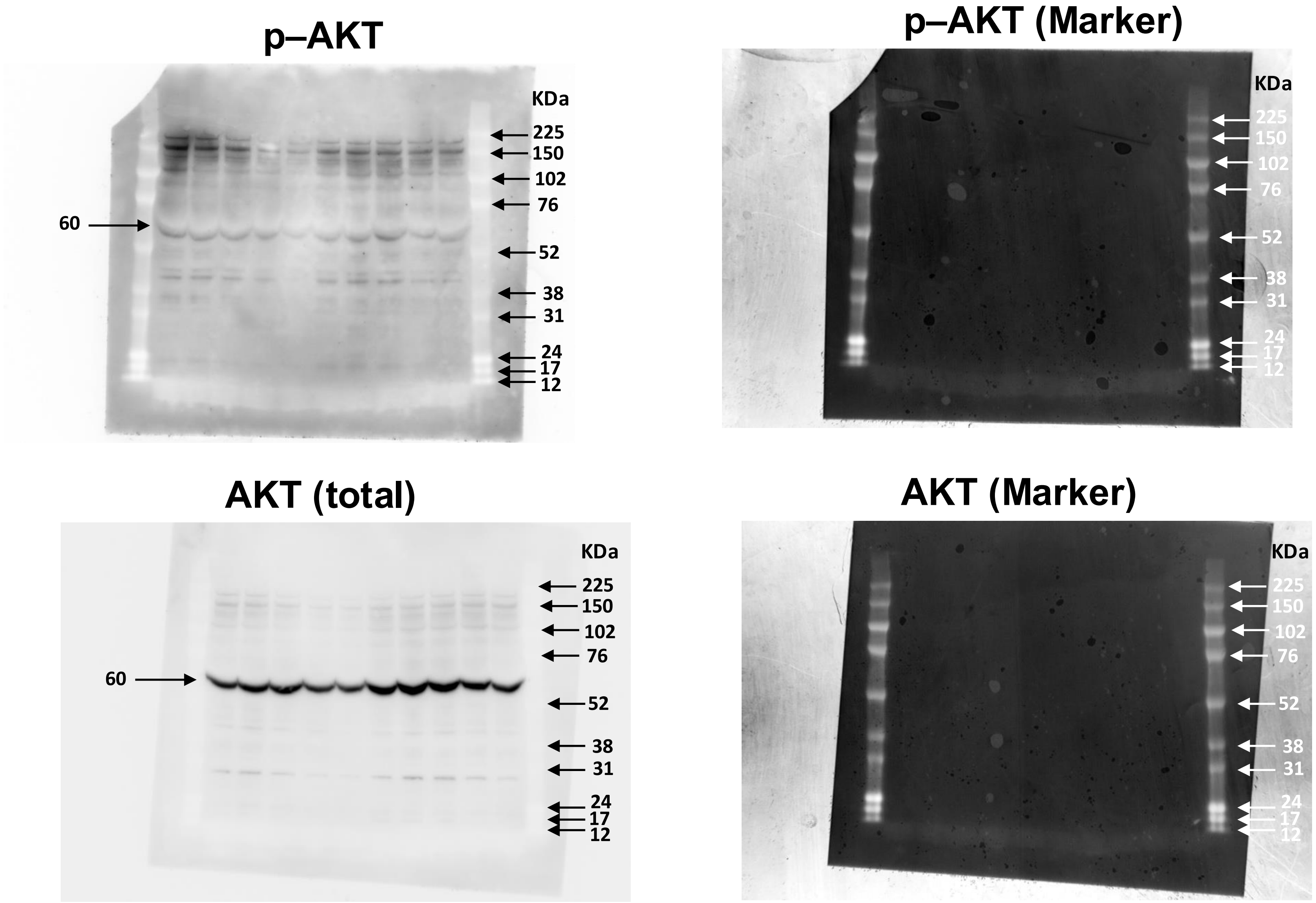

Supplemental Figure S20: Uncropped (raw) western blot images corresponding to Figure 1B showing p-AKT and AKT (total) expression in EPCs expressing NRAS^{WT} and NRAS^{Q61R}. Cells lysates were analyzed at 72 h after treatment with no doxycycline (−), doxycycline (+), or doxycycline (+) in combination with RMC−7977 (3.12, 12.5, 50 nM).

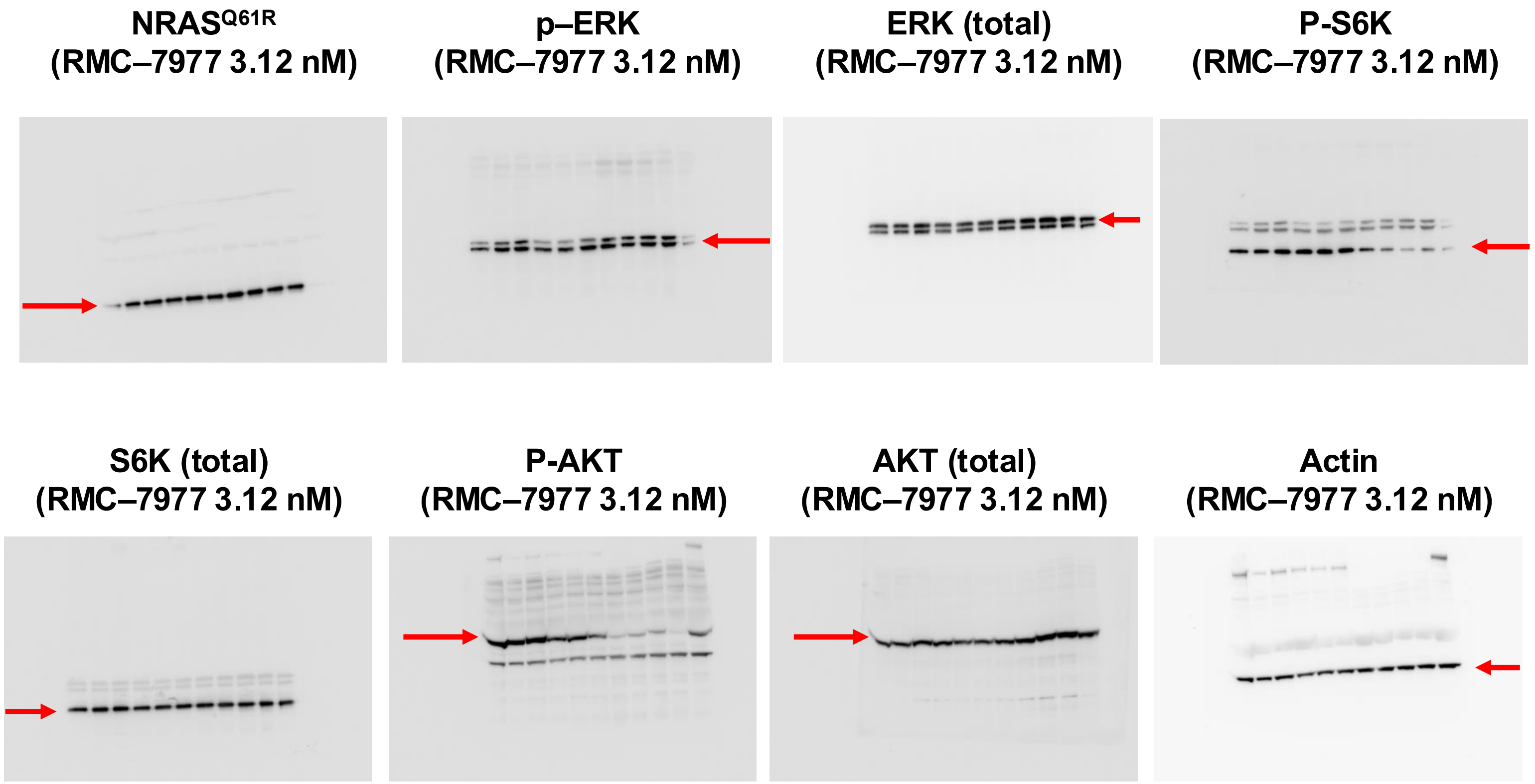

Supplemental Figure S21: Uncropped (raw) western blot images corresponding to Figure 1C (left) showing NRAS^{Q61R}, p−ERK, total ERK, p−S6K, total S6K, p-AKT, AKT (total), and actin expression in EPCs expressing NRAS^{Q61R}. Cells were treated with doxycycline for 48 h to induce NRAS^{Q61R} expression. Then media were changed with media containing no doxycycline (−), doxycycline (+), or doxycycline (+) in combination with RMC−7977 (3.12 nM). Cell lysates were collected at 0.5, 1, 3, 6, 24, 48, 72 h.

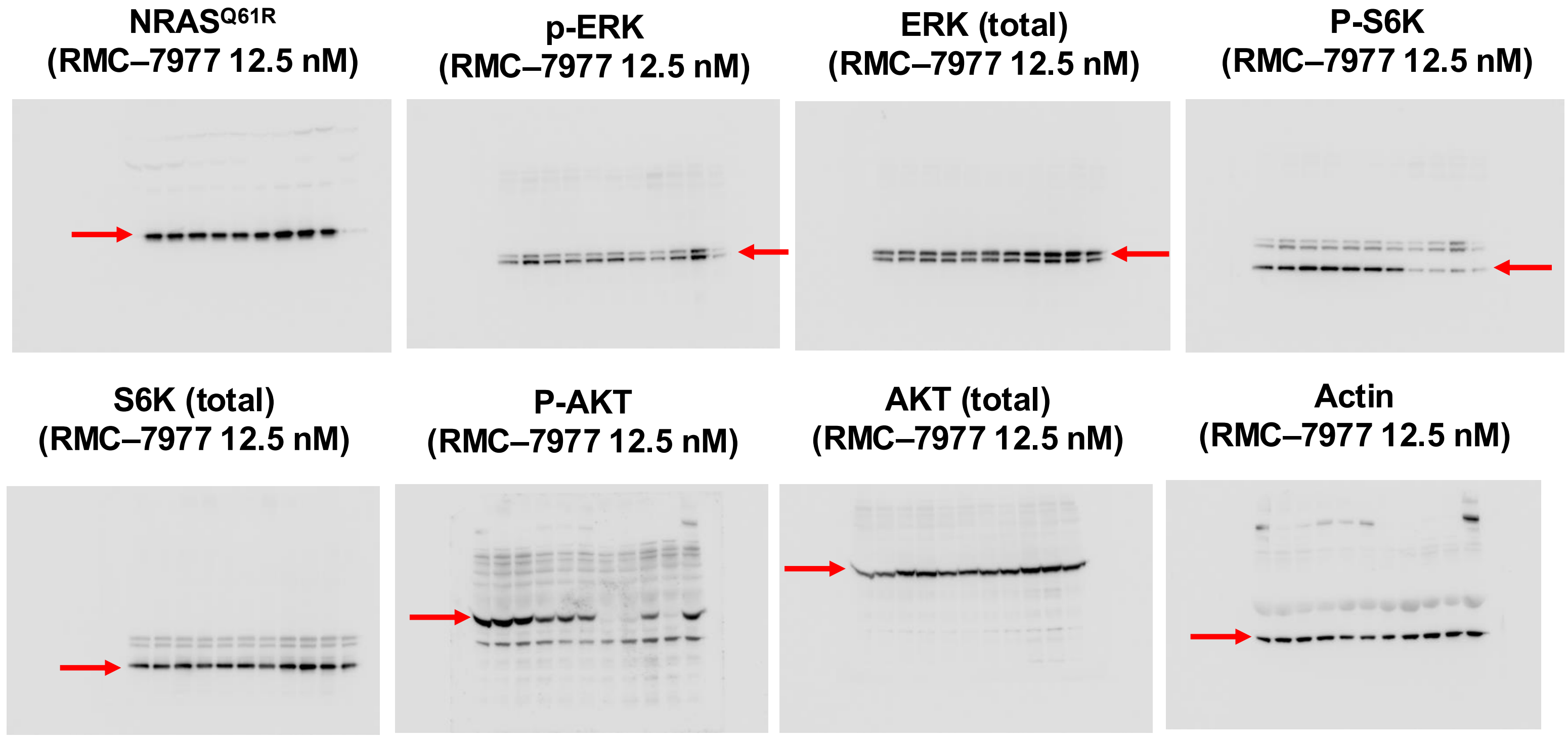

Supplemental Figure S22: Uncropped (raw) western blot images corresponding to Figure 1C (middle) showing $NRAS^{Q61R}$, p−ERK, total ERK, p−S6K, total S6K, p-AKT, AKT (total), and actin expression in EPCs expressing $NRAS^{Q61R}$. Cells were treated with doxycycline for 48 h to induce $NRAS^{Q61R}$ expression. Then media were changed with media containing no doxycycline (−), doxycycline (+), or doxycycline (+) in combination with RMC−7977 (12.5 nM). Cell lysates were collected at 0.5, 1, 3, 6, 24, 48, 72 h.

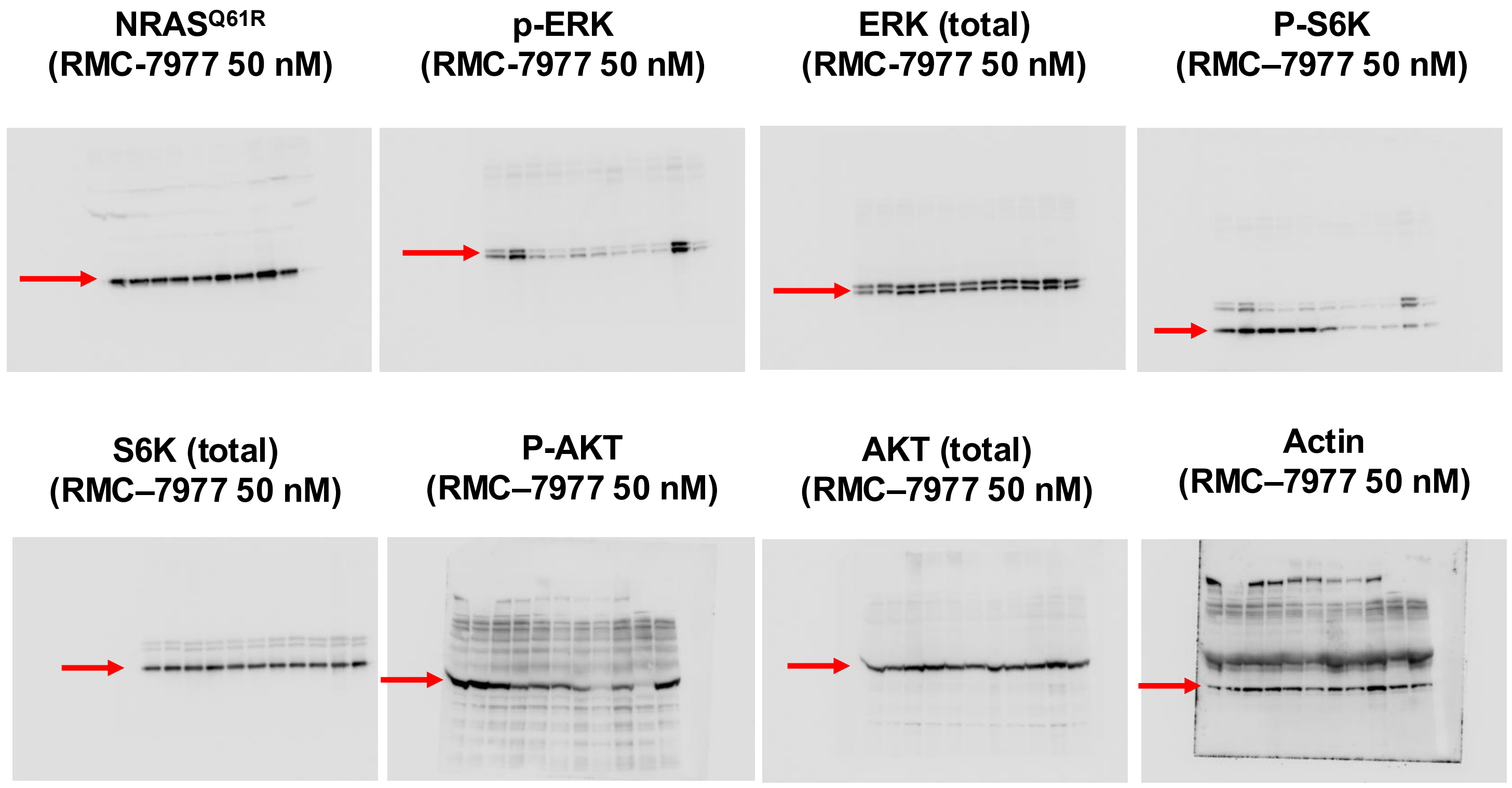

Supplemental Figure S23: Uncropped (raw) western blot images corresponding to Figure 1C (right) showing NRAS^{Q61R}, p−ERK, total ERK, p−S6K, total S6K, p-AKT, AKT (total), and actin expression in EPCs expressing NRAS^{Q61R}. Cells were treated with doxycycline for 48 h to induce NRAS^{Q61R} expression. Then media were changed with media containing no doxycycline (−), doxycycline (+), or doxycycline (+) in combination with RMC−7977 (50 nM). Cell lysates were collected at 0.5, 1, 3, 6, 24, 48, 72 h.

NRAS^{Q61R} Treated with RMC (3.12–100 nM) Dose Response Experiment

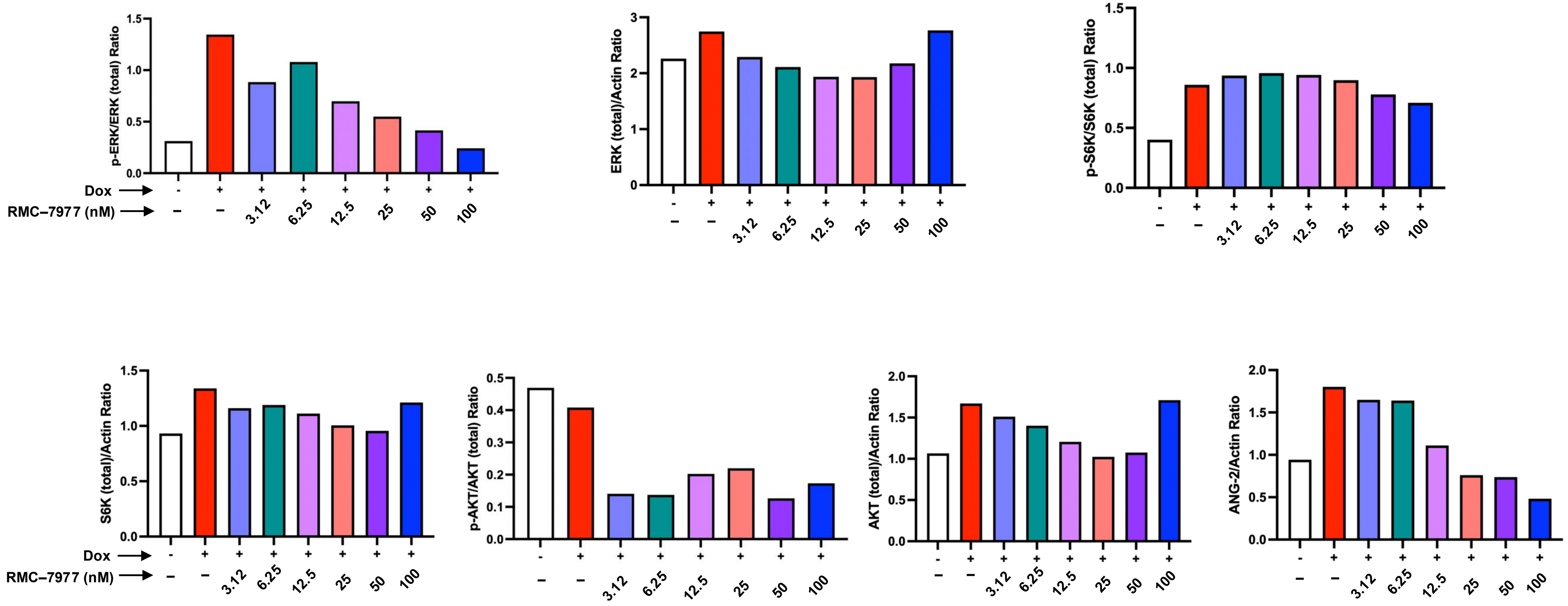

Supplemental Figure S24: Densitometric analysis of western blot shown in Figure 1A RMC-7977 Dose Response Experiment. Densitometric analysis of p-ERK/ERK, ERK/actin, p-S6K/S6K, S6K/actin, p-AKT/AKT, AKT/actin, ANG-2/actin protein expression in no doxycycline and doxycycline-induced NRAS^{Q61R} endothelial progenitor cells (EPCs) treated with increasing concentrations of RMC-7977 (3.12–100 nM) for 72 hours. Band intensities were quantified using Multi Gauge Software and normalized to total ERK, total S6K, total AKT, or actin.

$NRAS^{WT}$ and $NRAS^{Q61R}$ Treated with RMC (3.12, 12.5, 50 nM) Prevention Experiment

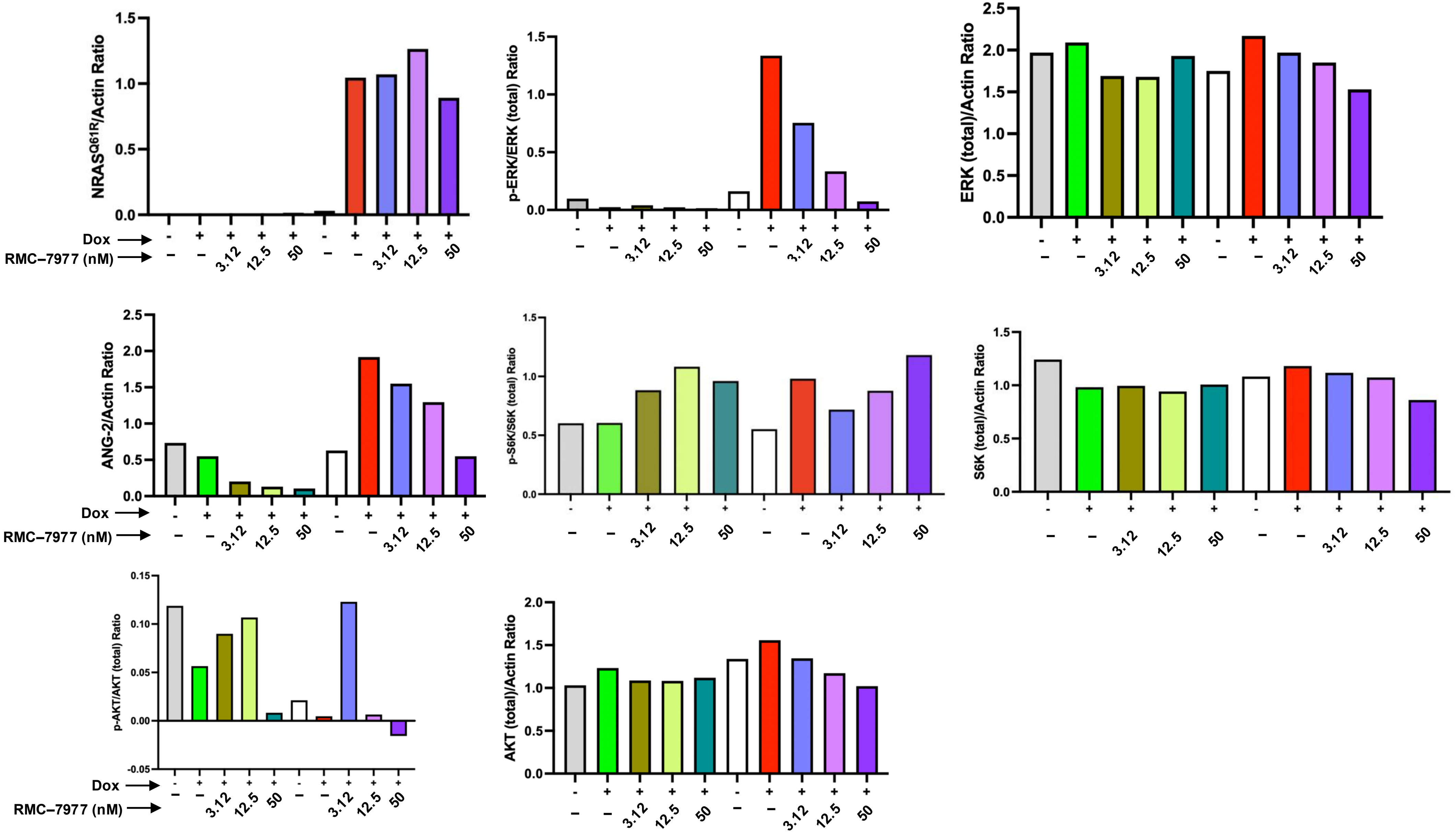

Supplemental Figure S25: Densitometric analysis of western blot shown in Figure 1B $NRAS^{WT}$ and $NRAS^{Q61R}$ Treated with RMC (3.12, 12.5, 50 nM) Prevention Experiment. Densitometric analysis of $NRAS^{Q61R}$/actin, p-ERK/ERK, ERK/ actin, p-S6K/S6K, S6K/actin, p-AKT/AKT, AKT/actin, ANG-2/actin protein expression in no doxycycline and doxycycline-induced $NRAS^{WT}$ and $NRAS^{Q61R}$ endothelial progenitor cells (EPCs) treated with increasing concentrations of RMC-7977 (3.12, 12.5, and 50 nM) for 72 hours. Band intensities were quantified using Multi Gauge Software and normalized to total ERK, total S6K, total AKT, or actin.

NRAS^{Q61R} treated with RMC 3.12 nM Time Course Analysis

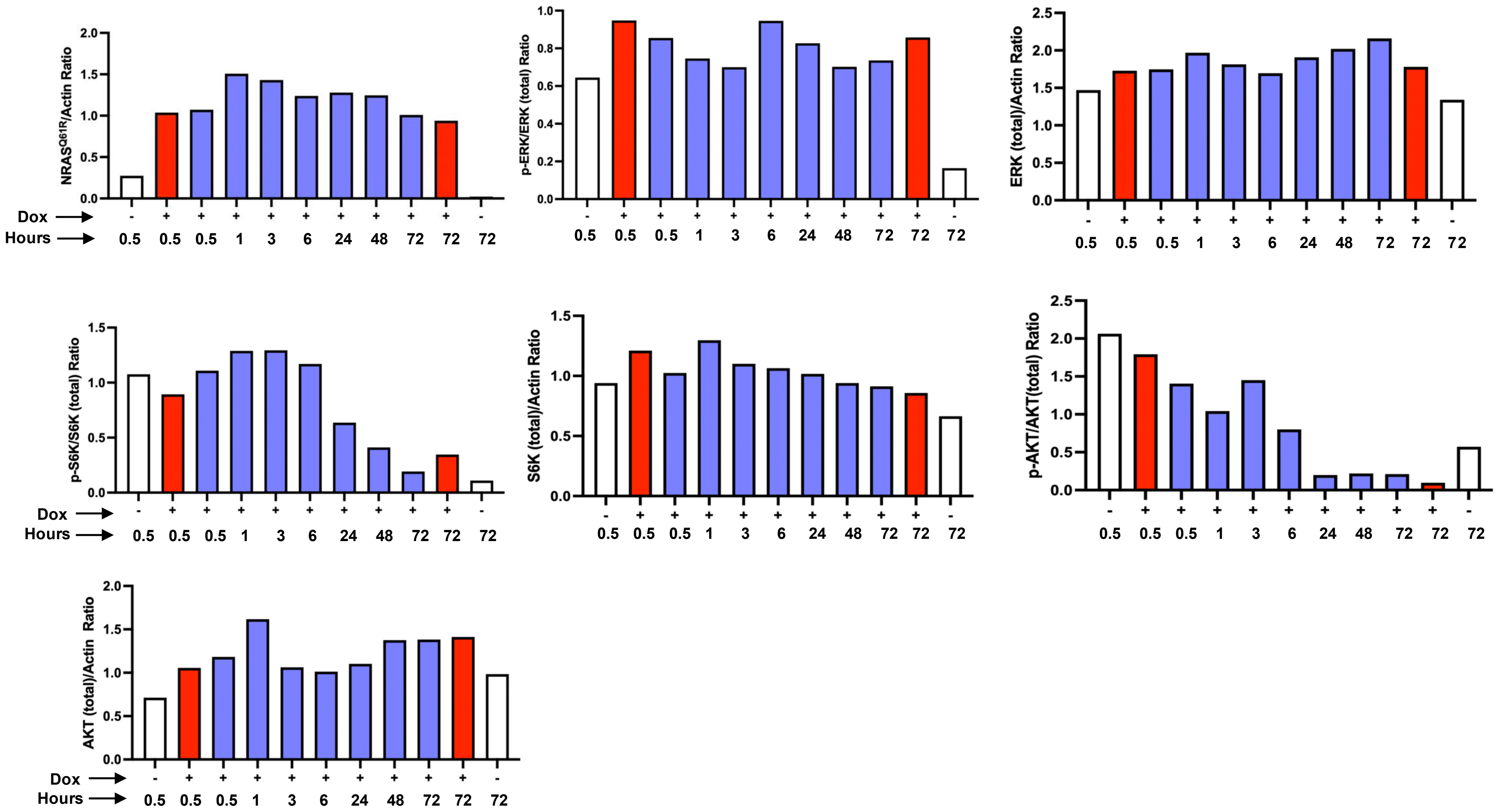

Supplemental Figure S26: Densitometric analysis of western blot shown in Figure 1C (RMC 3.12 nM) time course. Densitometric analysis of NRAS^{Q61R}/actin, p-ERK/ERK, ERK/ actin, p-S6K/S6K, S6K/actin, p-AKT/AKT, AKT/actin, ANG-2/actin protein expression. Cells were treated with doxycycline (Dox) for 48 h to induce *NRAS*^{Q61R} expression. The media was replaced with new media containing no doxycycline (−), doxycycline (+), and doxycycline (+) in combination with RMC−7977 (3.12 nM) added to the cell culture media for 0.5, 1, 3, 6, 24, 48, and 72 hours. Antibodies specific to p−ERK, total ERK, p−S6K, total S6K, p-AKT, and total AKT, and actin were used. Band intensities were quantified using Multi Gauge Software and normalized to total ERK, total S6K, total AKT, or actin.

NRAS^{Q61R} treated with RMC 12.5 nM Time Course Analysis

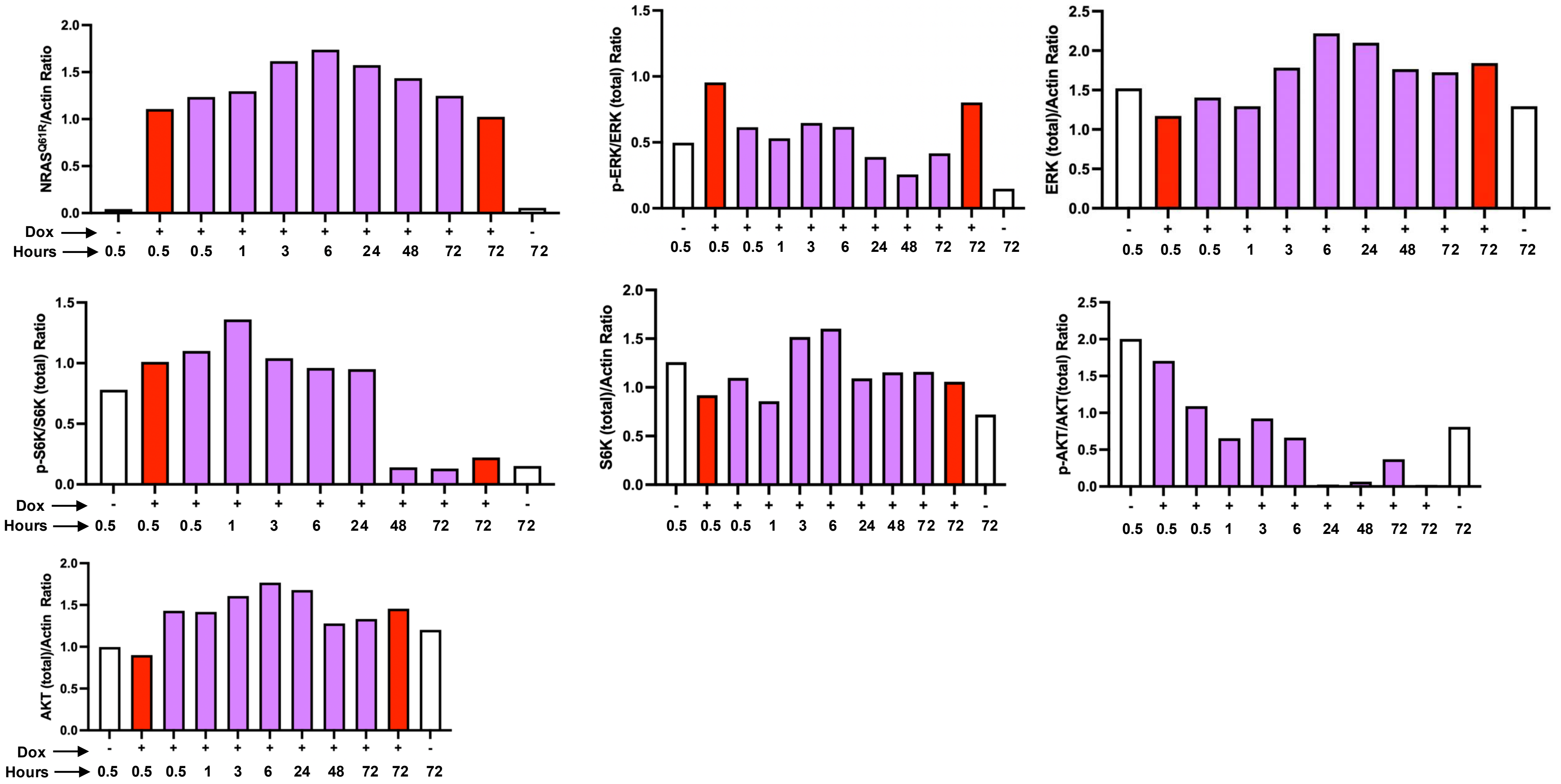

Supplemental Figure S27: Densitometric analysis of western blot shown in Figure 1C (RMC 12.5 nM) time course. Densitometric analysis of NRAS^{Q61R}/actin, p-ERK/ERK, ERK/ actin, p-S6K/S6K, S6K/actin, p-AKT/AKT, AKT/actin, ANG-2/actin protein expression. Cells were treated with doxycycline (Dox) for 48 h to induce *NRAS*^{Q61R} expression. The media was replaced with new media containing no doxycycline (−), doxycycline (+), and doxycycline (+) in combination with RMC−7977 (12.5 nM) added to the cell culture media for 0.5, 1, 3, 6, 24, 48, and 72 hours. Antibodies specific to p−ERK, total ERK, p−S6K, total S6K, p-AKT, and total AKT, and actin were used. Band intensities were quantified using Multi Gauge Software and normalized to total ERK, total S6K, total AKT, or actin.

NRAS^{Q61R} treated with RMC 50 nM Time Course Analysis

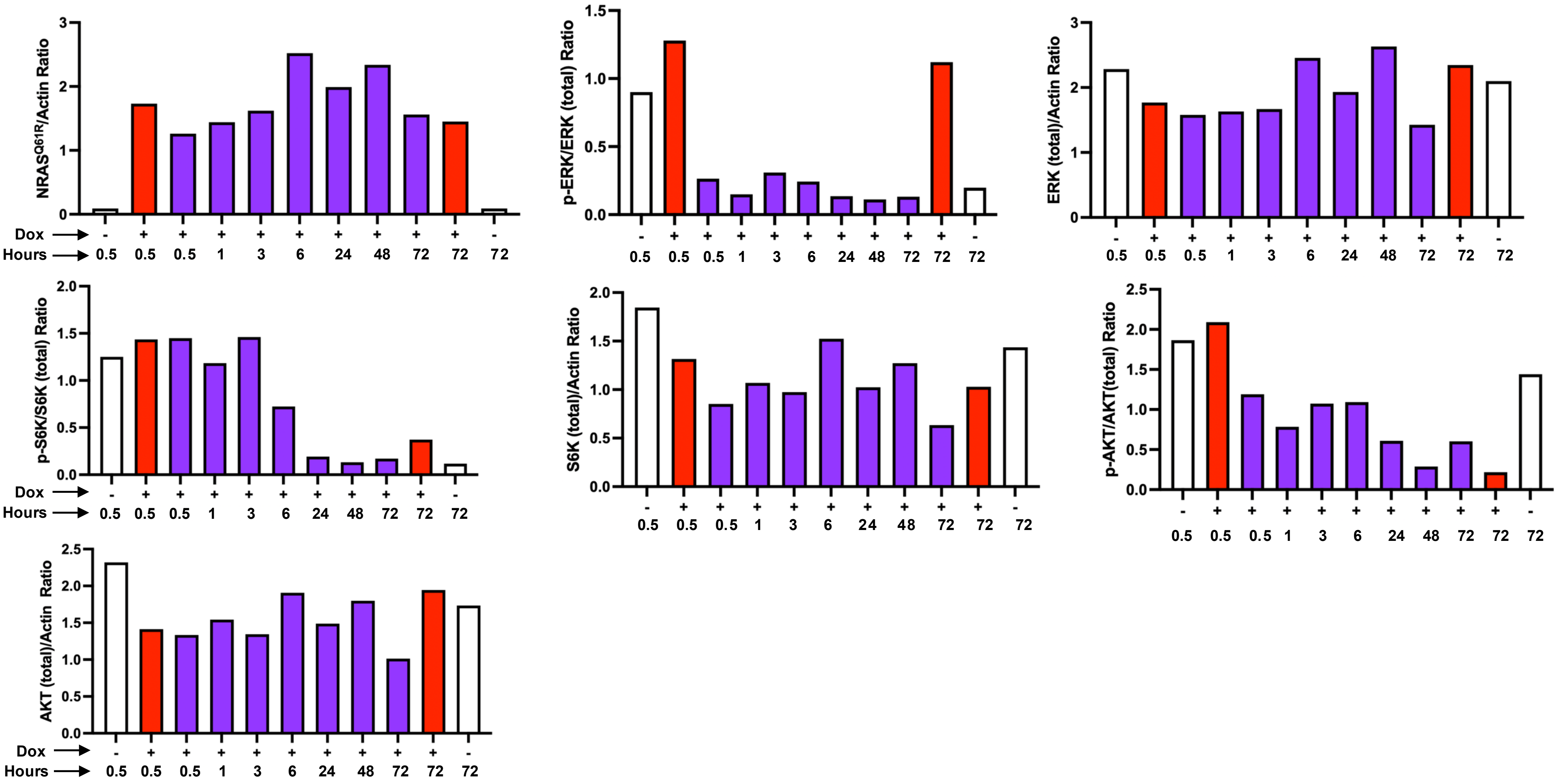

Supplemental Figure S28: Densitometric analysis of western blot shown in Figure 1C (RMC 50 nM) time course. Densitometric analysis of NRAS^{Q61R}/actin, p-ERK/ERK, ERK/ actin, p-S6K/S6K, S6K/actin, p-AKT/AKT, AKT/actin, ANG-2/actin protein expression. Cells were treated with doxycycline (Dox) for 48 h to induce *NRAS*^{Q61R} expression. The media was replaced with new media containing no doxycycline (−), doxycycline (+), and doxycycline (+) in combination with RMC−7977 (50 nM) added to the cell culture media for 0.5, 1, 3, 6, 24, 48, and 72 hours. Antibodies specific to p−ERK, total ERK, p−S6K, total S6K, p-AKT, and total AKT, and actin were used. Band intensities were quantified using Multi Gauge Software and normalized to total ERK, total S6K, total AKT, or actin.
