# Supplementary material for: RAS Inhibitor RMC‐7977 Blocks Vascular Overgrowth of NRASQ61R Mutant Endothelial Cells
Source: J Cell Mol Med. 2026 Aug 10;30(15):e71316. doi: 10.1111/jcmm.71316 (PMC13458280; doi:10.1111/jcmm.71316)
Supplement: Supplementary file 2 [file JCMM-30-e71316-s001.docx]

**Supplementary Table S1. Key Reagents for Western Blot and Staining**

| **Antibodies** | **Primary (dilution)** | **Vendor** | **Catalog Number** | **Secondary (dilution)** | **Vendor** | **Catalog Number** |
| --- | --- | --- | --- | --- | --- | --- |
| NRAS^Q61R^ | Rabbit recombinant monoclonal antibody  1:400 | Abcam | 227658 | Anti-Rabbit  (1:10,000) | Millipore Sigma | 401393 |
| ANG-2 | Polyclonal Goat IgG  (1:200) | R&D Systems | AF623 | Anti-goat (1:10,000) | Calbiochem | 401515 |
| p-ERK | Rabbit mAb (1:4000) in 5% BSA | Cell Signaling | 4370 | Anti-rabbit  (1:10,000) | Millipore Sigma | 401393 |
| ERK (total) | Rabbit mAb (1:1000) in 5% BSA | Cell Signaling | 9102 | Anti-rabbit  (1:10,000) | Millipore Sigma | 401393 |
| p-S6K | Rabbit mAb  (1:2000) in 5% BSA | Cell Signaling | 4858 | Anti-rabbit  (1:10,000) | Millipore Sigma | 401393 |
| S6K (total) | Rabbit mAb  (1:2000) in 5% BSA | Cell Signaling | 2217 | Anti-rabbit  (1:10,000) | Millipore Sigma | 401393 |
| p-AKT  (Ser473) | Rabbit mAb  (1:1000) in 5% BSA | Cell Signaling | 4060 | Anti-rabbit  (1:10,000) | Millipore Sigma | 401393 |
| AKT (total) | Rabbit mAb  (1:1000) in 5% BSA | Cell Signaling | 9272 | Anti-rabbit  (1:10,000) | Millipore Sigma | 401393 |
| Actin | Mouse  (1:10,000) in 5% non-fat dry milk | Seven Hills Bioreagents |  | Anti-mouse  (1:10,000) | Millipore Sigma | 401215 |
| Ulex europaeus Agglutinin I (UEA-1) | 1:100 | Vector Laboratories |  | Vector biotinylated goat a rabbit secondary |  |  |
| p-ERK (IHC staining) | 1:400 | Cell Signaling | 4370 | Vector biotinylated goat a rabbit secondary |  |  |
