# Supplementary material for: RAS Inhibitor RMC‐7977 Blocks Vascular Overgrowth of NRASQ61R Mutant Endothelial Cells
Source: J Cell Mol Med. 2026 Aug 10;30(15):e71316. doi: 10.1111/jcmm.71316 (PMC13458280; doi:10.1111/jcmm.71316)
Supplement: Supplementary file 3 [file JCMM-30-e71316-s002.docx]

**Supplementary Table S2. Key Reagents, kits, and plates for different experiments**

| **Reagents** | **Vendor** | **Catalog Number** |
| --- | --- | --- |
| Endothelial cell growth medium-2 (EBM2) | Lonza | CC-3156 |
| Endothelial cell growth medium-2 (EGM-2MV) | Lonza | CC-4147 |
| Fetal bovine serum (FBS) | Cytiva | SH30910.03 |
| 1% penicillin-streptomycin-glutamine | Fisher Scientific [Corning] | MT30002CL |
| Puromycin | Thermo Fisher Scientific [Gibco]) | A1113803 |
| Human Plasma Fibronectin Purified Protein | Millipore Sigma [Sigma-Aldrich] | FC010 |
| Doxycycline | Millipore Sigma [Sigma-Aldrich] | D3447 |
| RMC-7977 100 mg | Selleckchem | E1858 |
| PEG300 | MedChemExpress | HY-Y0873 |
| DMSO | Fisher Scientific [Fisher Chemical] | D128-500 |
| Tween™ 80 | MedChemExpress | HY-Y1891 |
| Saline | Baxter |  |
| Matrigel® | Millipore Sigma [Corning] | CLS354230 |
| Enzyme-linked immunosorbent assay (ELISA) | R&D Systems | DANG20 |
| 4%-12% Bis/Tris polyacrylamide gel | Thermo Fisher Scientific [invitrogen] | 24080771 |
| PVDF Membrane Filter Paper Sandwich 0.2um Pore Size | Thermo Fisher Scientific [life sciences] | 2929603 |
| Ibidi USA Culture-Insert 2 Well 24, #1.5 polymer coverslip | Fisher Scientific | NC2124782 |
| Cell culture/imaging microplate, black polystyrene with clear flat bottom 96-well | Agilent | 204626-100 |
| NucBlue™ Live ReadyProbes™ Reagent (Hoechst 33342) | Thermo Fisher Scientific | R37605 |
| Cytodex® 3 microcarrier beads | Cytiva | 17048501 |
| Fibrinogen | Sigma-Aldrich | F8630 |
| Aprotinin | Sigma-Aldrich | A1153 |
| Thrombin | Sigma-Aldrich | T4648 |
| Human dermal fibroblasts | ATCC | PCS-Z01-01Z |
